# Sonomechanobiology: Vibrational stimulation of cells and its therapeutic implications

**DOI:** 10.1063/5.0127122

**Published:** 2023-04-21

**Authors:** Lizebona August Ambattu, Leslie Y. Yeo

**Affiliations:** Micro/Nanophysics Research Laboratory, School of Engineering, RMIT University, Melbourne VIC 3000, Australia

## Abstract

All cells possess an innate ability to respond to a range of mechanical stimuli through their complex internal machinery. This comprises various mechanosensory elements that detect these mechanical cues and diverse cytoskeletal structures that transmit the force to different parts of the cell, where they are transcribed into complex transcriptomic and signaling events that determine their response and fate. In contrast to *static* (or *steady*) mechanostimuli primarily involving constant-force loading such as compression, tension, and shear (or forces applied at very low oscillatory frequencies ( ≤1 Hz) that essentially render their effects quasi-static), *dynamic* mechanostimuli comprising more complex vibrational forms (e.g., time-dependent, i.e., periodic, forcing) at higher frequencies are less well understood in comparison. We review the mechanotransductive processes associated with such acoustic forcing, typically at ultrasonic frequencies ( >20 kHz), and discuss the various applications that arise from the cellular responses that are generated, particularly for regenerative therapeutics, such as exosome biogenesis, stem cell differentiation, and endothelial barrier modulation. Finally, we offer perspectives on the possible existence of a universal mechanism that is common across all forms of acoustically driven mechanostimuli that underscores the central role of the cell membrane as the key effector, and calcium as the dominant second messenger, in the mechanotransduction process.

## I. INTRODUCTION

Tissue, bone, and organs continuously grow and remodel as part of the natural process by which living organisms develop and adapt to their environment and to therapeutic intervention.^1^ Central to these processes are cellular level responses to external physical, chemical, and biological cues. Although the role of mechanical forces constituting diverse physical cues that cells respond to in organogenesis has long been known,^1–3^ it has only been of late that the mechanostimulatory effects on physiological and pathological conditions have been increasingly studied, given their therapeutic implications, such as for nanomedicine and regenerative medicine.^2,3^

The mechanisms by which cells sense these mechanical forces and translate such cues into a cascade of cellular and molecular signaling events that ultimately determine their biological response and fate is, however, extremely complex.^2–4^ The majority of investigations in this area have primarily employed the application of static forms of mechanical forcing, be it compressional, tensile, or shear forcing,^4,5^ in which the applied loads were predominantly static, with the exception of a few “dynamic” studies,^5^ although these cases only involved mechanical forcing at very low repetition frequencies ( ≤1 Hz) such that the loads are essentially at constant force and the cells' mechanoresponse do not differ significantly from that observed for the steady, static cases.

Cells have nevertheless also been known to respond to vibration in which the applied force is time-dependent and essentially periodic, although this again has mostly been conducted at oscillation frequencies of several Hz—understandably given that this is the characteristic frequency associated with typical human or organism locomotion. More recently, though, it has been shown that more complex dynamic mechanical forcing in the form of acoustically driven vibrational stimuli, both at audible (20 Hz–20 kHz) and, curiously, even ultrasonic (20 kHz–1 GHz) and hypersonic ( >1 GHz), frequencies can also invoke a variety of cellular responses.

Existing reviews on mechanotransduction—the cellular process by which mechanical stimuli to the cell are transducted into biochemical signals^6–8^—have not just largely focused on static mechanical forcing but have also been primarily confined to their effects, in particular, on sensory cells. While this is because such cells function specifically to detect mechanical cues and are hence ideal models to study these effects,^8^ they do not capture the myriad of effects observed when other types of cells are mechanostimulated. There also generally appears to be a patchwork of investigations on different types of mechanostimuli with little attempt to understand the similarities or differences between the cellular responses and the underlying mechanotransduction mechanisms between them.

We, therefore, attempt to address this by providing a systematic perspective on the various cellular mechanostimulation strategies employing sound wave vibration that have been examined to date, together with their associated mechanisms, phenomena, and applications (touched on briefly by Rufo *et al.*^9^) particularly since this is considerably more complex and relatively less understood compared to their conventional mechanostimulation counterparts involving steady, constant-force compressional, tensile, or shear forcing. We begin in Sec. II by briefly revisiting the principles that underpin mechanotransduction. We then proceed to summarize the different forms of mechanostimuli that have been employed, beginning first with a brief overview of static constant-force mechanical loading in Sec. III, followed by a more extensive treatise on dynamic (periodic) mechanostimulation driven by sound wave vibration over a wide range of frequencies and the signaling pathways they induce in Sec. IV. We subsequently discuss their various applications, particularly for, albeit not limited to, nanomedicine and regenerative medicine (namely, cell-free and cell-based therapies, and cell/tissue reorganization) in Sec. V and show in Sec. VI that it is possible to adopt a unified mechanistic framework centered around membrane-tension-driven second messenger calcium-induced signaling that holistically captures the way different cells perceive various forms of acoustically driven mechanostimuli irrespective of the applied frequency and intensity.

## II. MECHANOTRANSDUCTION

**FIG. 1.**
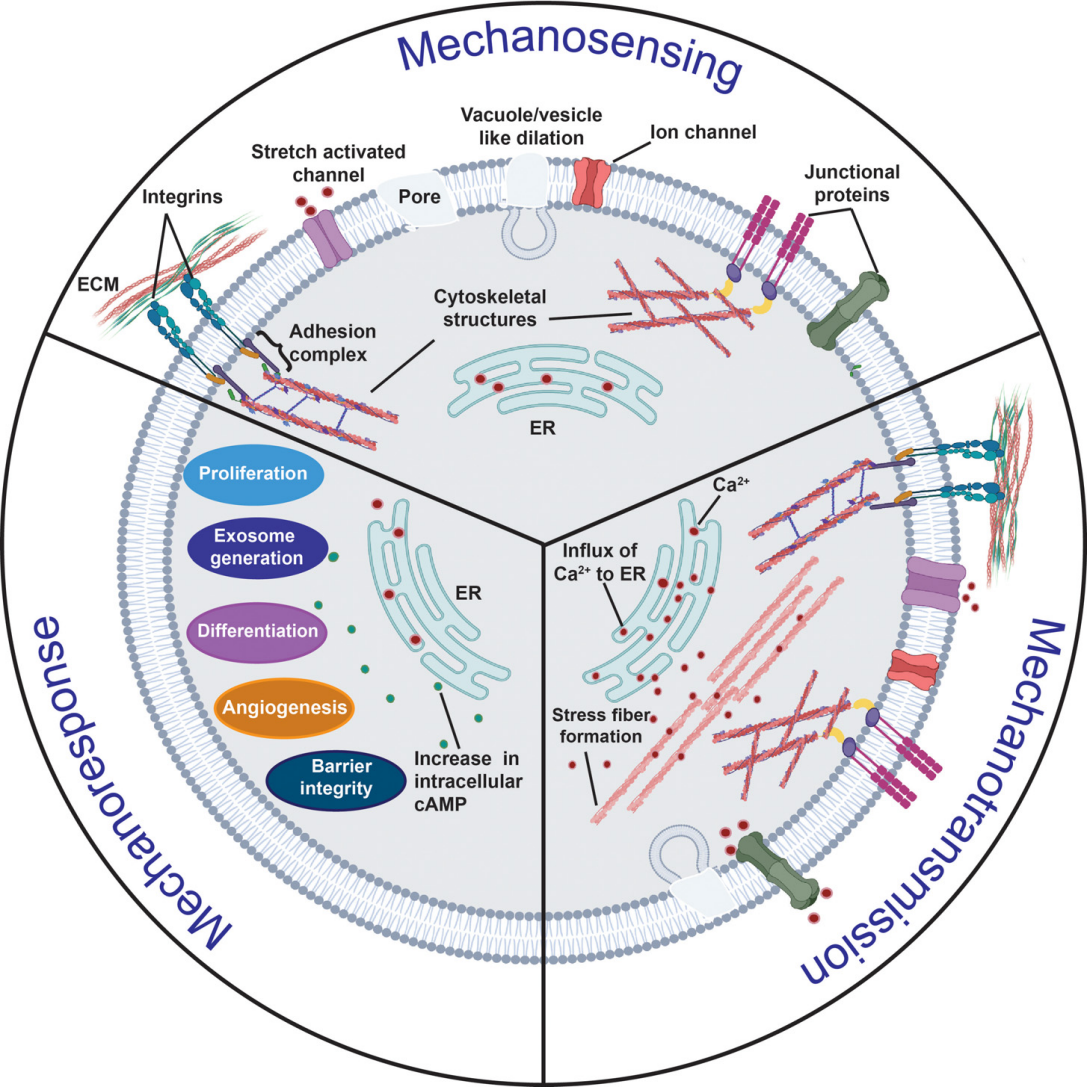
Overview of the mechanotransduction process in which a cell recognizes and responds to mechanical cues through three distinct stages. In the mechanosensing stage, the cell detects mechanical cues from the extracellular milieu primarily through local changes or perturbations along its plasma membrane, such as vacuole/vesicle-like dilations (VLDs) or pore formation, that arise as a consequence of the external force on the cell; alternatively, or additionally, other key mechanosensors such as transmembrane proteins (e.g., integrins) or junctional proteins [e.g., adherens junctions (AJs) such as cadherins, or, gap junctions (GJs) such as connexins] can also be involved in the process. In the mechanotransduction stage, the aforementioned conformational changes in the cytoskeletal structures associated with the mechanosensors as a result of the mechanical cues are then relayed to intracellular sensing structures; phosphorylation, ion transport (primarily Ca^2+^), and the activation of other second messengers, such as cyclic adenosine monophosphate (cAMP), also play a crucial role in the process. These transmitted cues subsequently trigger multiple downstream signaling cascades in the mechanoresponse stage, resulting in the activation of different cellular activities, such as cell migration, proliferation and differentiation, angiogenesis, exosome biogenesis, and barrier integrity maintenance.

Mechanotransduction—in which cells respond to mechanical stimuli over millisecond timescales^10^ associated with the time it takes for the cues to propagate to the nucleus^11,12^ where they are converted into autocrine, paracrine, or endocrine signals, which, in turn, trigger downstream local or global events that maintain homeostasis or direct cellular fate—is a particularly complex phenomenon. At a fundamental level, the mechanotransduction process can be viewed as comprising three distinct stages involving mechanosensing, mechanotransmission, and mechanoresponse steps (Fig. 1). In simpler models, these are considered to be in series, although more complex descriptions allow for dynamic coupling between the stages to account for regulation of the signaling pathways by the applied mechanical load.^13^

### A. Mechanosensing

There are two categories of mechanosensors that facilitate cellular perception of a force applied to it through a change in either their molecular assembly or a conformational change in its protein structure: passive (outside–in), wherein the mechanosensing element detects an external force imparted on it and transmits the signal into the cell, or, active (inside–out), in which forces are generated by the mechanosensing element to detect changes in the extracellular matrix (ECM).^6,14^ Various mechanosensory elements exist in the form of proteins embedded at the cell–ECM interface that are sensitive to membrane stiffness changes [e.g., focal adhesions (FAs), membrane receptor proteins such as G-protein coupled receptors, adherens junctions (AJs), and stress activated channels (SACs)], all of which are involved in either or both passive or active sensing.

FA protein complexes under the cell membrane, for example, play a role in both forms of mechanosensing by constituting the bridge between the ECM and the intracellular domain. This connectivity occurs through the recruitment of transmembrane reporters known as integrins, whose intracellular tails are connected to the actin cytoskeleton by resident adaptor proteins, such as vinculin and talin, and whose extracellular head domains bind to proteins in the ECM^6^—the latter being regulated by the mechanical stimuli, which induces a conformational change, i.e., unbending of integrins that result in the exposure of the binding sites.^15^

**FIG. 2.**
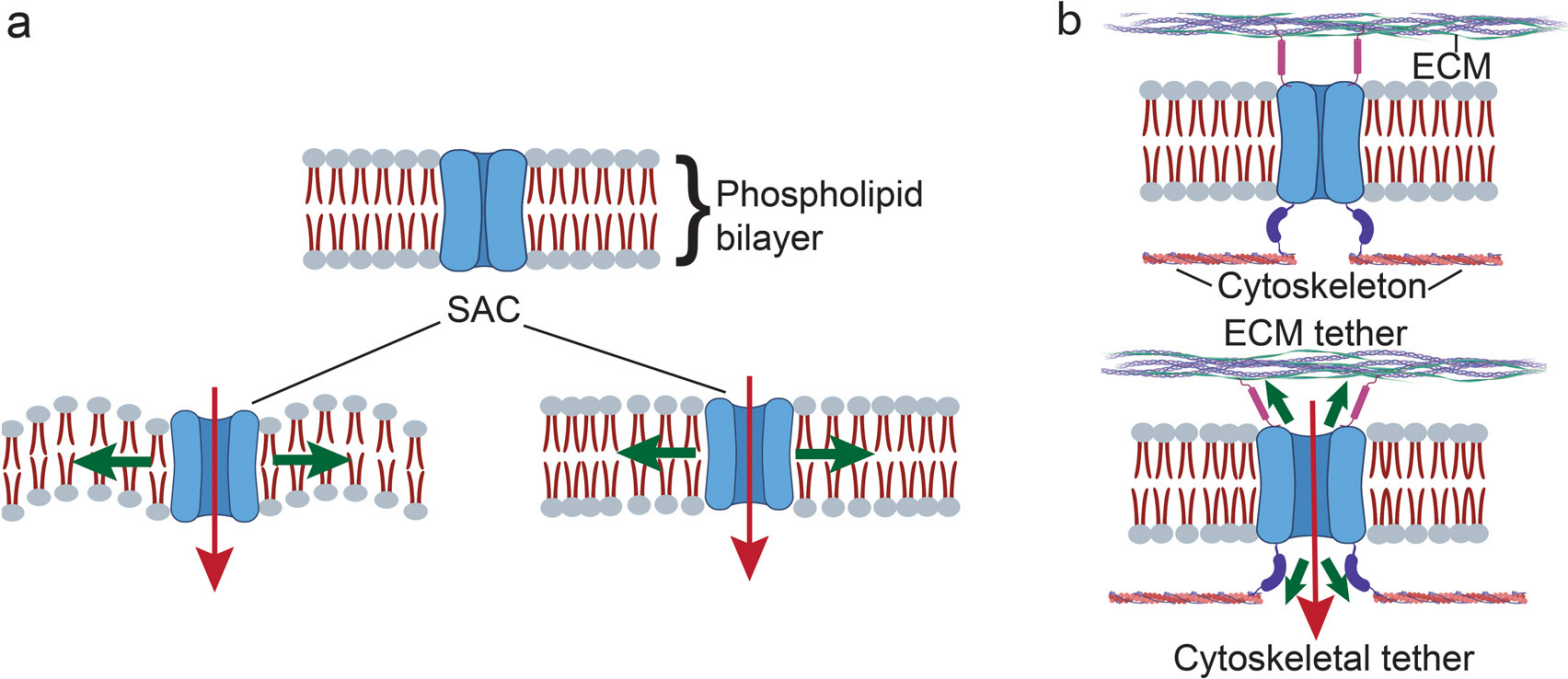
Two accepted models for SAC gating. (a) In the force-from-lipid (FFL) model, the reorganization of the lipid bilayer structure of the cell membrane in response to the applied mechanical loading exerts a force on the SAC to regulate its gating. (b) In the force-from-filament (FFF) model, the SACs are tethered to the extracellular matrix (ECM) or the cytoskeleton, through which the force is exerted.

SACs or mechanosensitive ion channels, which facilitate gating of the ion transport in and out of the cell, constitute another dominant type of mechanosensor,^16^ especially given the role of calcium (Ca^2+^) as a key second messenger and signaling molecule that is critical to the dynamic triggering of downstream transcriptomic changes;^17^ intracellular Ca^2+^ concentrations in particular also play a central role in regulating a wide range of cellular processes.^17^ Two different models exist to describe the gating mechanism by which SACs operate (Fig. 2). In the stretch model [also known as the force-from-lipid (FFL) model^18^], tension imposed by any mechanical perturbation to the cell membrane leads to the reorganization of the lipids in the bilayer around the transmembrane SACs as well as a conformal change in its proteins that results in the opening of the channel.^19^ The tether, or force-from-filament (FFF), model, on the other hand, suggests that the SACs are physically connected directly to the ECM or indirectly through intracellular accessories such as the cytosekeleton.^20^ These tethers then interact directly with channel or auxiliary proteins, indirectly imposing tension on the channel and allowing for long-range propagation of membrane tension, therefore facilitating long-distance sensing via the cytoskeletal structure.^20–22^

In eukaryotes, four main types of SACs have been identified: two-pore-domain (K2P) potassium channels, degenerin–epithelial sodium–acid-sensing ion channels (DEG–ENaC–ASIC), transient receptor potential (TRP) channels, and piezo channels.^23^ The K2P family of ion channels consists of 15 members (KNCK genes), which can be distributed within six subfamilies: TWIK (tandem of pore domains in a weak inward rectifying K^+^ channel), TREK (TWIK related K^+^ channels), TASK (TWIK related acid-sensitive K^+^ channels), TALK (TWIK-related alkaline pH-activated K^+^ channels), THIK (tandem pore domain halothane inhibited K^+^ channels), and TRESK (TWIK related spinal cord K^+^ channel).^24^ DEG–ENaC–ASIC channels in vertebrates comprise two subgroups: ENaC and ASIC; while ENaC is known to be sensitive to osmotic swelling, shrinking, and shear stress, the mechanosensitivity of ASIC has not as yet been fully explored.^25^ The TRP channel family, on the other hand, consists of 28 channels that are grouped into 6 subfamilies, namely, canonical TRP (TRPC), vanilloid TRP (TRPV), melastatin-related TRP (TRPM), ankyrin TRP (TRPA), mucolipin TRP (TRPML), and polycystic TRP (TRPP).^26,27^

The gating mechanisms involved in all of these ion channels, and, in particular, the DEG–ENaC–ASIC channels, are not completely understood.^28^ However, it is likely that membrane tension plays a key role in controlling the opening and closing of these channels, although we note that the factors initiating the gating through membrane tension varies between each subfamily. For instance, TREK and TRAAK channels were found to be sensitive to shear stress, negative membrane pressure, and temperature,^29^ whereas TRP channels are known to be insensitive to membrane tension induced by cell membrane stretching.^30^ The TRPV, TRPM, and TRPA subfamilies, in contrast, are thermally activated, while exocytosis was found to activate the TRPC, TRPV, and TRPM channels.^27^

Piezo channels—vertebrate mechanically activated nonselective cation channels—are a more recently discovered member of the SACs family,^31^ whose role has been widely implicated in mechanosensing. Whereas Piezo1 initiates Ca^2+^ signals predominantly in various non-excitable cells,^31^ Piezo2 mainly functions in sensory neurons and specialized cell types, such as tactile epithelial cells (e.g., Merkel cells^31^). Piezo channels can be activated by membrane tension (force-from-lipid), indicating that any physiological force that can cause indentation of the cell membrane on the apical surface-lumen facing side and hence alter the membrane tension, such as that produced with a blunt glass probe or suction to the membrane, can robustly activate the piezo channel.^32^ It has also been noted that mechanical perturbations imposed onto the basolateral surface of the membrane, for instance, those at the cell–cell and cell–matrix interfaces produced by stretching cells seeded on tensile substrates or on elastomeric pillar arrays, or by compressing the membrane using an atomic force microscopy (AFM) probe, can also activate these channels, thereby alluding to the key role that piezo channels play in sensing of the microenvironment.^33^

Both localized and whole-cell responses can be mediated through Piezo1 regardless of the endogenous or exogenous origin of the mechanical stimulus.^34^ As long-range propagation of membrane tension in intact cell membranes is limited, long-distance sensing is enabled through biochemical and functional tethering of the piezo channel to the actin cytoskeleton via the mechanotransduction complex comprising the AJ complex (cadherin and β-catenin) and cytoskeletal-associated proteins (vinculin), which allow complementary force-from-filament gating via the cytoskeletal network.^34^

Upon their activation, piezo channels generate cationic nonselective currents; the dominant cation that arises is however dependent on the cell type.^35^ For example, piezo channel activation in cells such as sensory neurons leads to membrane depolarization owing to Na^+^ influx, with Ca^2+^ playing a minor role,^36^ whereas Ca^2+^-induced signaling cascades are elicited in endothelial cells (ECs)^37^ and mesenchymal stem cells (MSCs).^38^ As such, piezo channels have been recognized to possess different roles along diverse mechanotransduction pathways depending on the physiological environment that they are exposed to.

### B. Mechanotransmission

A hierarchy of load-bearing cytoskeletal structures, such as F-actin, intermediate filaments, and microtubules, are critical for continuous propagation of the force across the entire length of the cell, either from within it, i.e., the nucleus, through the perinuclear space to the ECM, or vice versa. Such transmission occurs over time scales on the order of a tenth of a second^10^ and is regulated by a “molecular clutch” mechanism in which the size of the FAs along which the signals are transmitted are dependent on the applied force.^39^ Key interactions implicated in the transmission of force across the cell include myosin-induced actin contraction and molecular bond (e.g., actin–adaptor proteins, adaptor protein–integrin, or integrin–ECM) formation and dissociation;^13^ the rate of the latter determining the efficiency of transmission.^13^

### C. Mechanoresponse

**FIG. 3.**
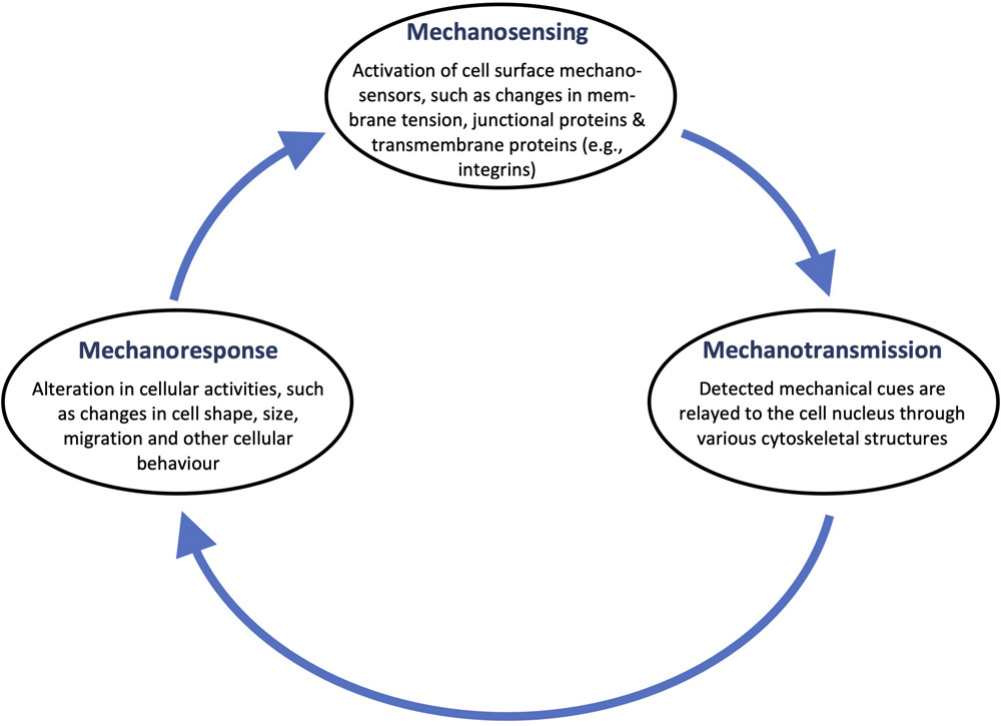
The closed-feedback-loop nature of the mechanotransduction process. Changes in mechanosensors are relayed to the nucleus, which alter various cellular activities and behavior. These include changes to cell shape and size, as well as the propensity of the cell to migrate, which, in turn, can act as a trigger for further sensing to maintain cellular homeostasis.

The final mechanotransduction stage, which involves processes such as calcium signaling, phosphorylation cascades, and DNA transcription factor binding encompasses the ability of the cell to transcribe the detected and transmitted mechanical cues into complex transcriptomic and signaling events. It is quite typical that the entire mechanotransduction process comprises a transcription feedback loop (Fig. 3) in which the cells' response is often transmitted back to the mechanosensitive structures that first produced the response.^13,40^

**FIG. 4.**
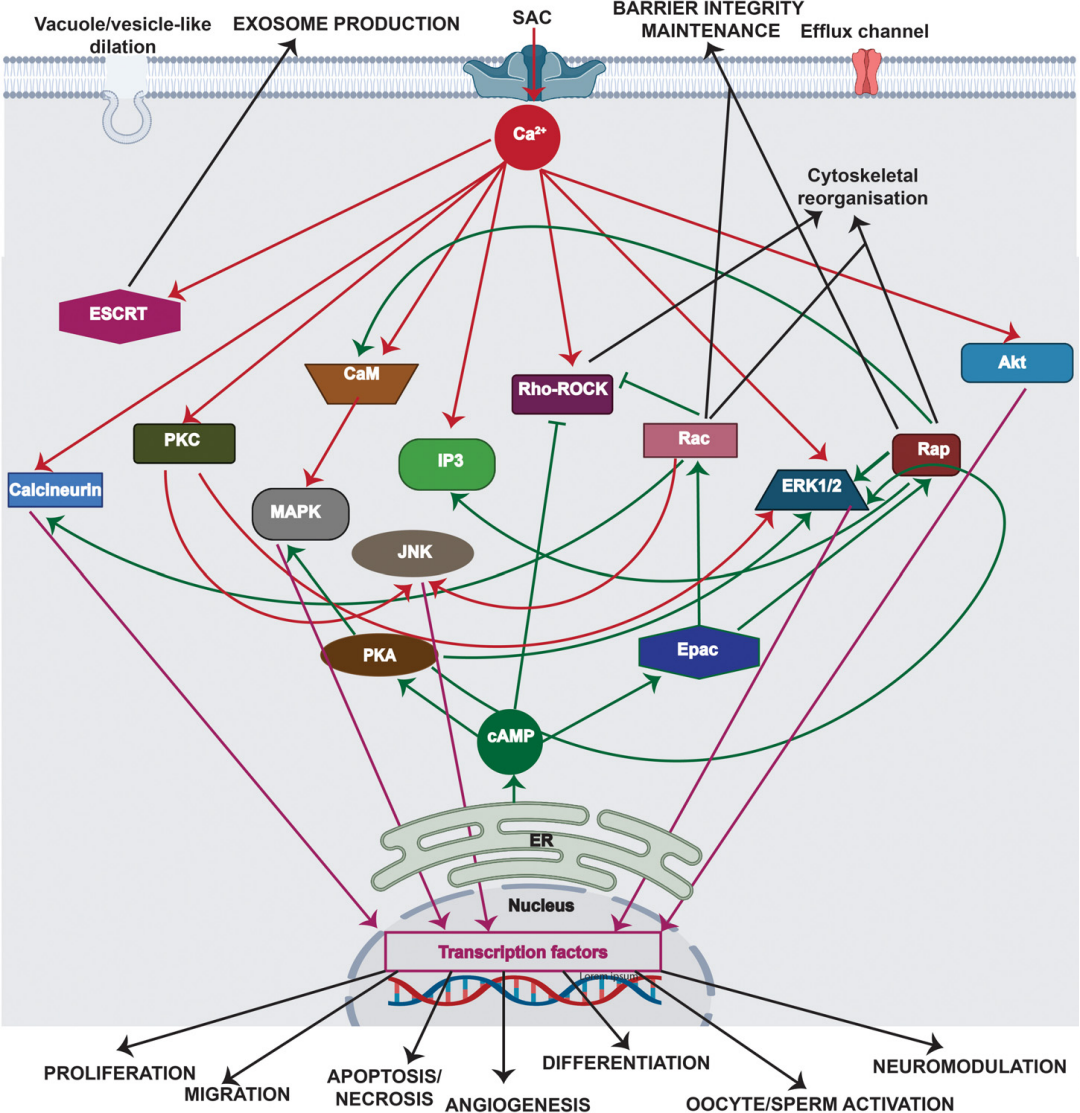
Major signaling cascades associated with the mechanotransduction process (for simplicity, only the key pathways involved in mechanotransduction are shown). These can be broadly categorized into Ca^2+^-facilitated pathways (red lines) and cAMP-assisted pathways (green lines) that either result in transient (e.g., cytoskeletal reorganization, barrier integrity maintenance, or exosome production) or permanent (e.g., cell migration, proliferation, apoptosis, angiogenesis and differentiation, or cellular activation or neuromodulation) changes in cell behavior. Transient changes can be initiated by phosphorylation of substrates associated with the Rho–ROCK proteins and through ESCRT pathways. Permanent changes, on the other hand, can be initiated by signaling cascades, such as the MAPK, JNK, ERK1/2, or Akt pathways, which activate transcription factors, such as c-Fos, c-Jun or CREB, that are triggered by both Ca^2+^ and cAMP. The normal arrows → depict the activation of signaling cascades, whereas the inhibitory arrows ⊣ denote inhibitory effects. (Rho: Ras homologous protein; ROCK: Rho-associated protein kinase; MAPK: mitogen-activated protein kinase; ESCRT: endosomal sorting complexes required for transport; Rap: Ras-related protein; PKC: protein kinase C; CaM: calmodulin; IP3: inositol 1,4,5-trisphosphate; Rac: Ras-related C3 botulinum toxin substrate; ERK: extracellular signal-regulated kinase; Akt: protein kinase B; cAMP: cyclic adenosine monophosphate; Epac: exchange factor directly activated by cAMP; PKA: cAMP-dependent protein kinase; c-Fos: AP-1 transcription factor subunit; c-Jun: transcription factor AP-1; CREB: cAMP responsive element binding protein; JNK: c-Jun N-terminal protein kinases).

Unlike mechanosensing and mechanotransmission, which occur rapidly on the order of milliseconds, mechanoresponses take place over significantly longer time scales: the activation of signaling pathways, for example, occurs immediately over minutes, whereas the induction of gene expression pathways is typically delayed and occurs over hours or days.^41^ Depending on the magnitude and duration of the imposed mechanical cue, these delayed responses can lead to transient changes in vesicle trafficking or cellular morphology, permanent compositional or structural alterations in adhesion complexes and cytoskeletal structures, and/or the triggering of secondary signaling cascades that facilitate cell migration or proliferation, cancer progression, angiogenesis, or differentiation.^3,42^ Key mechanoresponses are depicted in Fig. 4.

## III. STATIC OR QUASI-STATIC CONSTANT-FORCE MECHANOSTIMULATION

As alluded to in Sec. II, cells have the ability to sense and respond to both exogeneous as well as endogenous forces. The literature to date has therefore found it convenient to broadly delineate these into extrinsic and intrinsic stimuli—the former constituting external forces physically exerted on the cell, and the latter being the cues “felt” by the cell from its surrounding environment, namely, the ECM. Both are known to regulate cell function. In reality, however, both extrinsic and intrinsic cues are intricately interrelated since mechanotransduction is essentially bidirectional, wherein nuclear mechanoresponses are transmitted back to the mechanosensors and cytoskeletal structures at the cell–ECM interface to give rise to a feedback loop, as discussed above. As such, the cell response to intrinsic stimuli cannot, therefore, be easily or simply decoupled from its extrinsic counterpart. Nevertheless, such a categorization provides a simple and convenient framework to understand the effects of different stimuli on cell fate.

Given our focus in this review on external mechanostimulatory cues, and, in particular, those arising through acoustic forcing, we refer the reader to the excellent articles that provide extensive commentary on intrinsic stimulations.^40,43–46^ Briefly, intrinsic stimulation in the cell can arise due to changes to its shape and density, ECM, or substrate topology.^40^ Altering the cell shape and density, both of which are interrelated, has, for example, long been known to affect cell spreading and proliferation: densely seeded cells tend to assume a rounder shape, whereas low seeding densities allow cells to spread and grow.^47,48^ In stem cells, this was reported to promote osteogenesis over adipogenesis.^49^ Restricting the cell shape through geometrical confinement or through the introduction of topology, for instance, through micropatterning of the substrate on which the cells are grown, on the other hand, can lead to apoptosis.^50,51^ Cues arising from changes to the mechanical property of the ECM (e.g., stiffness and viscoelasticity), on the other hand, are detected through the FA adhesion complex that anchors the cell to ECM proteins;^52^ the accompanying conformation change in the integrin-binding domains (Sec. II A) is relayed to the cytoskeletal complex, triggering a signaling cascade involving the Rho family of small GTPases (RhoA, Rac and Cdc42) that are responsible for cytoskeletal organization. These, along with their downstream effectors, Rho-associated protein kinase (ROCK), myosin light chain (MLC) kinase and yes-associated protein (YAP), or transcriptional coactivator with PDZ binding motifs (TAZ), are known to induce cell migration and cell cycle progression apoptosis, or to direct stem cell differentiation.^40^

**FIG. 5.**
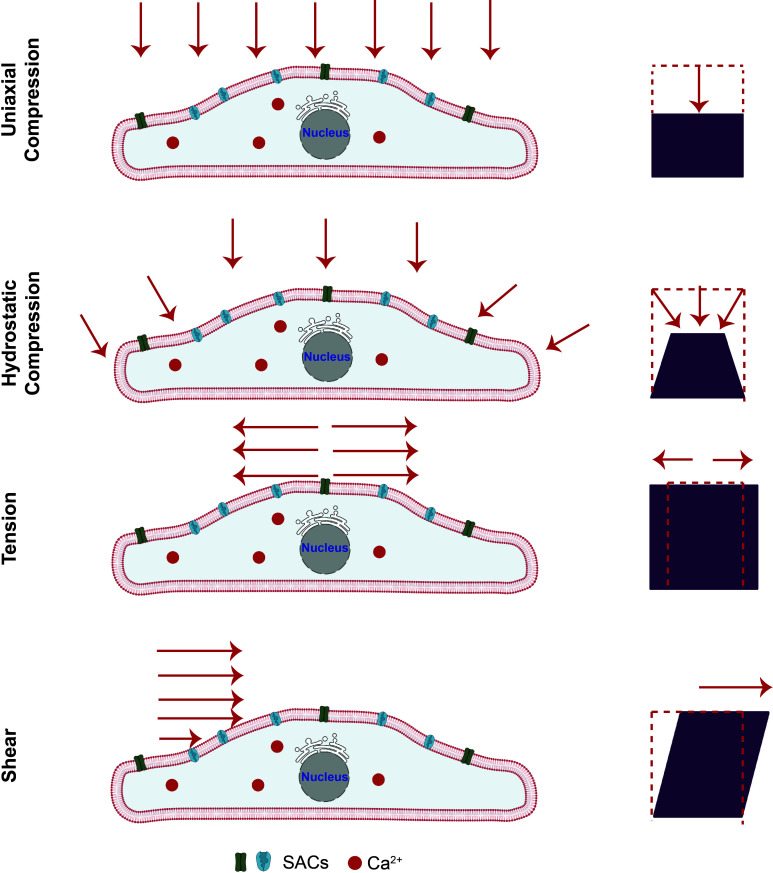
Static forms of mechanostimulation. Schematic depiction of different extrinsic mechanical load, which can induce compression (uniaxial compression or hydrostatic compression), tension or shear stress, on the cells. Adapted from Ref. 40.

For extrinsic mechanostimulation, the bulk of work carried out to date has primarily involved the application of static or steady forms of mechanical loading under compression, tension, or shear (Fig. 5). Periodic forcing, typically in the form of oscillatory vibrations or sound waves, has only been more recently explored and constitutes the subject of the present review. We nevertheless first provide here an overview of the existing work involving constant-force mechanical loading (Table I) to provide the reader with a contextual basis toward a better understanding of the more complex dynamic nature of their time-dependent (periodic forcing) counterpart to be discussed subsequently.

**TABLE I.** The different forms of mechanostimuli and their key mechanosensing and mechanoresponsive pathways.

Mechanostimulation	Relevant mechanosensors	Key mechanoresponsive signaling pathways
Compression	Integrins,^53^ piezo channels,^54,55^ TRP channels,^54,55^ FAs^53^	Wnt/ β-catenin,^56^ YAP/TAZ^57^
Tension	Integrins,^58,59^ piezo channels,^60^ TRP channels,^61,62^ FAs,^63–65^ and cytoskeletal elements^66^	Rho–ROCK,^66^ MAPK,^63^ and ERK^63^
Shear	Integrins,^62,67^ TRP channels,^62,68^ FAs,^69^ G-protein and G-protein receptors,^70^ tyrosine kinase receptors,^71,72^ lipid rafts,^73,74^ primary cilia,^75^ and GJs^76,77^	Rho–ROCK,^78,79^ MAPK,^80^ ERK,^81^ and Wnt/ β-catenin^82,83^
Acoustic stimulation	Piezo channels,^84^ TRP channels,^85^ FAs,^86^ AJs,^87^ and GJs^88^	Rho–ROCK,^84^ MAPK,^89^ ERK,^90^ Wnt,^91^ ESCRT,^92^ Epac–Rap,^87^ YAP,^93^ and PI3K/Akt^94^

### A. Compression

In studies to date, cells have generally been subjected to two different forms of compressive loading: hydrostatic compression/pressure and direct uniaxial compression.^95,96^ In the former, compression is applied to the media, and hence, the applied pressure on the cells is isotropic, whereas directly compressing the cell-laden scaffold uniaxially in the latter maintains the applied force in a specific direction. Unlike direct compression, hydrostatic compression/pressure can initiate volumetric membrane deformation without shear induced deformation.^97^ Yet, despite this difference, both forms of compression appear to induce similar mechanosensing and mechanotransmission mechanisms involving integrins, FAs, SACs, and Ca^2+^-signaling. In particular, compression induces distortion of cell surface receptors and integrins, through which the signal is transferred along the FA complex via the involvement of both vinculin and mature tight junctions.^53,98^ Additionally, mechanosensing through SACs such as piezo and TRP channels has also been implicated in both forms of compression to drive an influx of Ca^2+^ in the cell, and, in some cases, activation of actin-related protein (ARP2/3) complexes, which, in turn, mediates actin remodeling within the cell.^54,55^

Compression-induced downstream signaling is, however, not well understood to date,^57^ although YAP and TAZ signaling are among suggested downstream cascades induced by compressive mechanostimulation,^57^ which, in turn, affect the cells' ability to proliferate and undergo apoptosis, although these effects are largely dependent on the cell type.^97,99–103^ Additionally, compressive loads along with biochemical factors (interleukin-6) have also been shown to promote ECM remodeling while mediating epithelial-to-mesenchymal transition.^56^ In adipocytes, compression was observed to trigger Wnt/ β-catenin signaling that, in turn, induces dedifferentiation of the cells, resulting in a distinct transcriptome profile, long-term self-renewal, and serial clonogenicity (including osteogenesis, adipogenesis, myogenesis, chondrogenesis, and myofibrogenesis).^104^

### B. Tension

Numerous techniques have been developed to mechanically stretch a cell. These differ primarily based on the type of actuation scheme used to generate the tensile load.^105^ Tensile stretching has been reported to influence cell polarizaion/migration, morphology, proliferation, lineage commitment, and differentiation.^106,107^ The exact response that is observed, however, depends on both cell type^108^ as well as the mode by which the stretch is generated, in addition to the properties of the ECM and the presence of soluble factors.^109–112^ Major stretch-induced mechanotransduction signaling elements include integrins, force-sensitive kinases and proteins, cytoskeletal elements,^66^ and SACs,^60–62^ although the exact mechanism by which stretch-induced mechanotransduction occurs has not yet been clearly elucidated due to the complexity of the various processes and their dependence on a number of different parameters.^58,59,63–65^ In all cases though, the stretched-induced stimuli are relayed into the cells through a change in intracellular Ca^2+^ signaling. In particular, it was reported that mechanically stretched cells exhibited intracellular Ca^2+^ oscillations through SAC activation, which is manifested in F-actin assembly, actomyosin contractility, and associated Rho–ROCK signaling;^66^ the downstream effector of these being the mitogen-activated protein kinase (MAPK) and extracellular signal-regulated kinase (ERK)-induced signaling cascades. Similarly, various stretch-induced signaling cascades have been found to initiate osteoblast differentiation and regulation of morphogenesis, angiogenesis, heart remodeling, and neuritogenesis.^113–115^

### C. Shear

There have been a number of studies on the mechanotransductive effect of shear given its prevalence in physiological conditions where cells in the endothelium are constantly subjected to such stresses arising as a consequence of vasculatory flow. Studies employing flow channels to investigate the response of ECs under flow over a range of exposure durations from seconds to hours have now also been extended toward smaller scales to mimic conditions in the microvasculature with more recent advances in microfluidics.^116^

At the fundamental conceptual level, shear stress involves the activation of various intracellular and extracellular mechanosensors, such as transmembrane integrins,^62,67^ ion channels and SACs (in particular, TRP channels),^62,68^ G-proteins and their receptors,^70^ tyrosine kinase receptors,^71,72^ lipid rafts,^73,74^ and primary cilia,^75^ among others. More specifically, shear stresses and their gradients have been reported to upregulate α_v_ with β integrin subunits^117–119^ and their association with FAs, force–modulated through lipid raft diffusion,^69^ and with the adaptor protein Shc.^118^ Shear flow is also known to drive upregulation in GJs, as well as the activation of TRP channels, to allow modulation of cations, particularly Ca^2+^,^76,77^ which, in turn, results in the triggering of important downstream signaling pathways. These, depending on cell type, include, although are not limited to, the Rho–ROCK pathway in osteoblasts and osteocytes,^78,79^ the ERK1/2 MAPK pathway in stem cells^80,81^ and ECs,^120,121^ or the Wnt/ β-catenin pathway in ECs and fibroblasts,^82,83^ all of which play a role in modulating multiple cellular activities.^122–128^

## IV. ACOUSTICALLY DRIVEN MECHANOSTIMULATION: TIME-DEPENDENT (PERIODIC) VIBRATIONAL FORCING

Unlike the static forms of steady mechanical loading discussed in Sec. III, acoustic forcing provides an alternative and versatile means for cell stimulation. In addition to the ability of harnessing different forms of sound waves, the periodic nature of the vibrational forcing introduces further possibilities for dynamic stimulation that can be tuned over a broad range of applied frequencies, as will be discussed below.

Sound, in essence, is a mechanical wave generated by a vibrating surface or object. It mainly propagates through an elastic media as longitudinal (pressure; vibration displacement parallel to the direction of wave propagation) waves that involve the compression and rarefaction of the molecules in that medium. As it is possible to support vibrations in other directions in solids, transverse (shear) waves can also exist where the vibration displacement is transverse to the direction of wave propagation.

The sound wavelength λ is related to the frequency *f* by the speed at which the sound wave propagates through the medium *c*, which is dependent on its density and elasticity. Given this relationship and the broad spectrum of sound frequencies—ranging from infrasound ( <20 Hz), audible (20 Hz–20 kHz), ultrasound (20 kHz–1 GHz) to hypersound ( >1 GHz)—together with the different configurations of sound wave generation devices, different wave modes—i.e., the different ways sound waves can propagate through the media—can arise.^129,130^

**FIG. 6.**
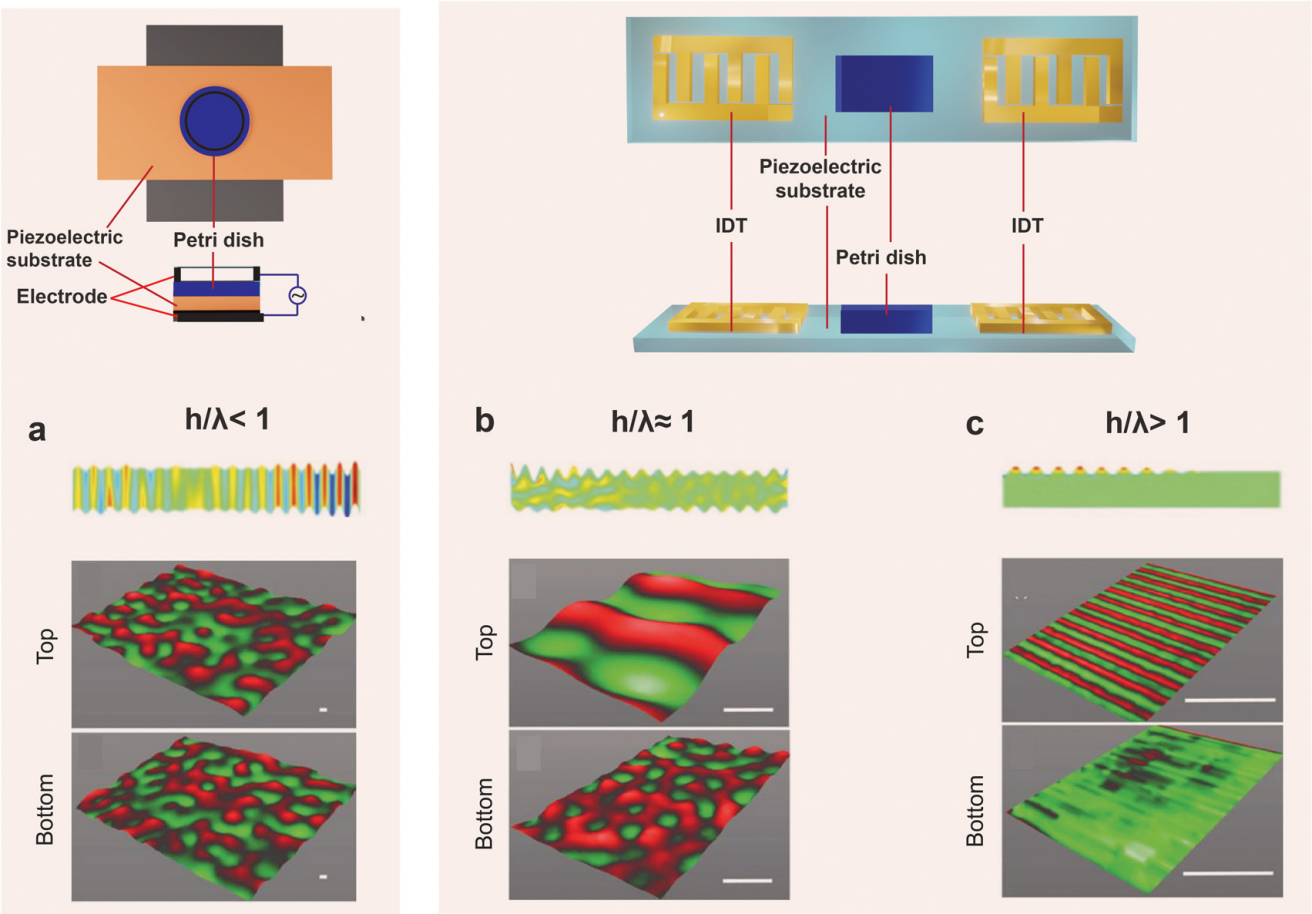
The different sound wave modes and the way in which they are generated on piezoelectric substrates: (a) bulk acoustic waves (BAWs), (b) hybrid surface and bulk acoustic waves [known as surface reflected bulk waves (SRBWs)^131^], and (c) surface acoustic waves (SAWs). The top row comprises top and side view sketches illustrating how these different wave forms are generated on piezoelectric (often, lithium niobate is used) substrates: (a) BAWs are generated by applying an AC electric field through a conducting layer or strip on the top and bottom surfaces of the piezoelectric substrate, whereas (b) SRBWs and (c) SAWs are typically generated using the same setup in which interdigitated transducer (IDT) electrodes are patterned on the piezoelectric substrate to which the AC electric field is applied; the thickness of the substrate *h* relative to the wavelength λ and hence resonant frequency f=c/λ is what determines which wave form is generated: (a) BAWs usually arise when h/λ<1, (b) SRBWs when h/λ≈1, and (c) SAWs when h/λ>1.^131^ The bottom row shows actual laser Doppler vibrometry scans measuring the displacement velocity on the top and bottom surfaces of the piezoelectric substrate for each case. Reproduced in part with permission from Rezk *et al.*, Adv. Mater. **28**, 1970 (2016). Copyright 2016 Wiley-VCH GmbH & Co. KGaA.

Bulk acoustic waves [BAWs; Fig. 6(a)] do not only take the form of longitudinal (pressure) or transverse (waves). Thin solid sheets with thicknesses h<λ (or h/λ<1), for example, can support plate waves that propagate parallel to its surface and through the thickness of the material. If the plate is infinitely wide, only a thickness mode exists, whereas a plate with finite width gives rise to symmetric (extensional) or asymmetric (flexural) Lamb waves that comprise both thickness and width modes. Surface acoustic waves [SAWs; Fig. 6(c)], which are also known as Rayleigh waves, in contrast, occur in piezoelectric substrates whose thicknesses are much greater than the acoustic wavelength, i.e., h>λ or h/λ > 1, and supports a combination of both longitudinal and transverse waves. A hybrid surface and bulk wave known as surface reflected bulk waves [SRBWs or pseudo-SAWs; Fig. 6(b)], on the other hand, can also exist in the intermediate transition regime where h≈λ or h/λ ≈ 1.^131^

In practice, the coupling of sound waves to laboratory cell cultureware can be achieved with the use of a transducer. At low frequencies in the infrasound and audible range, longitudinal bulk acoustic waves can be induced in a culture chamber by coupling the piston-like vibration generated with a conventional sound transducer akin to that found in loudspeakers. To generate BAWs at ultrasound frequencies up to several MHz, piezoceramic transducers are typically used, on which electrode pads are patterned [alternatively, higher frequency (MHz) BAWs can also be generated on piezoelectric substrates, as illustrated in Fig. 6(a)^132^]. The thickness of the transducer or substrate then determines its resonant frequency, and hence, the frequency of the bulk waves that are produced. While it is possible to generate bulk vibration at higher frequencies beyond several MHz, this requires considerably thinner resonant structures or typically thin piezoelectric film resonators with thicknesses *d* that are equal to half the resonant wavelength λ (i.e., the resonant frequency f=c/2d, wherein *c* is the sound speed in the piezoelectric film) mounted atop either an air gap (thin film bulk acoustic resonators, or FBAR) or multiple reflection layers (solid mounted resonators, or SMR).^133^

For SAWs and SRBWs, a chipscale piezoelectric substrate is employed, on which interdigitated transducers (IDTs)—electrodes patterned in an interleaved pattern—are photolithographically patterned, whose gap and spacing determines the resonant frequency of the device and hence the wavelength and frequency of the SAW or SRBW that propagates along the substrate [Figs. 6(b) and 6(c)]. Whether an SRBW [Fig. 6(b)] or SAW [Fig. 6(c)] is generated depends on this resonant frequency and hence wavelength relative to the substrate thickness.^131^

### A. Bulk waves at infrasound and audible frequencies ( <20 kHz)

There has only been limited work to date on mechanostimulation using bulk waves at infrasound ( <20 Hz) and audible (20 Hz–20 kHz) frequencies. This is likely because of the relative ease by which it is possible to generate other mechanical stimuli at Hz-order frequencies (which are the frequencies typically experienced by cells in their local physiological environment during human motion, e.g., walking and running), for example, using oscillatory versions of mechanical forcing such as the laminar shear or stretching discussed in Sec. III. Similarly, it is relatively simple to excite cells at ultrasonic frequencies at tens to hundreds of kHz given decades of progress in ultrasound technology, borrowing from the extensive advances from imaging and diagnostic and imaging modalities, which will be discussed in Sec. IV B.

Most of the work on acoustic cell stimuli in the infrasound and audible ranges has primarily been limited to cell proliferation,^134–138^ cell migration,^138,139^ and even apoptosis.^85^ Neuronal degeneration has also been reported in astrocytes, neurons, and cardiomyocytes exposed to 16 Hz infrasound,^85,140^ whereas upregulation in neuronal differentiation was observed with 1 kHz mechanostimulated bone marrow-derived stem cells (BMD-SCs),^141^ although we note that the latter appeared to be dependent on the inclusion of specific biochemical additives, such as dexamethasone, ascorbate, β-glycerophosphate, bone morphogenetic protein (BMP), 3-isobutyl-1-methylxanthine, insulin, transferrin, valproic acid, forskolin, neural supplement and human transforming growth factor β1, among others.^142^

It should be noted though that even for acoustic stimulation in the same frequency range, the mechanoresponse varies considerably between cell types. NIH/3T3 mouse immortalized fibroblasts and NB2a mouse neuroblastoma cells, for example, were found to be insensitive to audible sound wave excitation over the range 55 Hz–4 kHz, whereas genes such as prostaglandin-endoperoxide synthase 2 (Ptgs2)—an osteoblastic differentiation gene, connective tissue growth factor (CTGF), and ECM protein tenascin-C (TNC) were observed in ST2 (mouse stromal) and C2C12 (mouse immortalized myoblast) cells, indicating that while normal cells may be responsive to acoustic mechanostimulation, cancer cells, and immortalized cells (depending on the tissue source) are resistant to bulk vibrational stimuli, at least in the audible range.^143^

In any case, the majority of these studies do not explicate the mechanotransductive mechanisms responsible for these responses, although some have noted the involvement of intracellular Ca^2+^.^85,141^ More specifically, upregulation in TRPV4 (transient receptor potential cation channel subfamily V member 4) expression has been observed, which led to an increase in intracellular Ca^2+^ concentration in cells exposed to infrasound at 16 Hz for more than 7 days.^85^ Increases in intracellular Ca^2+^ concentration in astrocytes at this frequency have also been attributed to the opening of GJ proteins (connexin-43).^136^ Such Ca^2+^-induced signal transduction has been noted to activate the calmodulin and protein kinase C pathways, resulting in the release of glutamate^136^ or pro-inflammatory cytokines, both of which result in neuronal death.^140,144^ Similarly, ryanodine receptor (RyR)-induced Ca^2+^ signaling has also been reported in non-auditory cells such as MSCs when stimulated by audible sound waves (1 kHz),^141^ leading to the alteration of the cytoskeletal structure that, in turn, initiated a downstream chain of mechanotransductive events,^139,143^ primarily those related to intracellular Ca^2+^-induced signaling cascades, such as the ERK pathway.^141^

### B. Low frequency ultrasonic bulk waves (20 kHz–3 MHz)

The main bulk of the studies on vibrationally induced mechanotransduction has been carried out using bulk sound wave excitation at tens to hundreds of kHz. For convenience, we have opted to use the terminology “low frequency ultrasound” to refer to sound wave excitation at frequencies in this range, specifically from 20 kHz to approximately 3 MHz, which is typically the upper limit at which BAWs at ultrasonic frequencies can be generated.^145^ We have done so to distinguish this from the “high frequency ultrasound” mechanostimulation work carried out above this frequency range from several MHz up to 1 GHz that will be discussed in Sec. IV C given the considerable mechanistic differences that arise between them. Not least, high frequency ultrasonic excitation, in contrast, typically comprises surface or hybrid modes (SAWs or SRBWs), as introduced in the preamble of this section.

In the medical ultrasound imaging literature, low frequency ultrasonic bulk wave excitation has commonly been viewed to interact with biological matter through thermal and non-thermal effects.^146^ At the cellular level, thermal effects, which arise as a consequence of the dissipation of sound energy as heat upon its absorption in tissue, can be extremely detrimental to cells, which typically denature above 40 °C cells due to hyperthermia.^147–150^ Stem cells are even more sensitive to thermal effects and suffer from heat shock with temperature rises of merely 4 °C (even within the physiological range).^151^ Non-lethal thermal effects, on the other hand, include the release of heat shock proteins,^152^ and altered protein conformation, leading to misfolding or nonspecific aggregation of proteins, which result in cell cycle arrest or impaired growth rates.^147^ Further, temperature variations can give rise to changes in the cell membrane tension, that, in turn, triggers the activation of ion channels such as TRP (particularly TRPV)^27^ and K2P channels (TREK and TRAAK),^29^ which alters the intracellular Ca^2+^ concentration and potentially instigates various downstream signaling cascades.^152^ As such, it is extremely important to control the intensity as well as the duration of the mechanostimulation to minimize these adverse effects on cells, which can also include poor reseeding viabilities and colony-forming ability,^153^ although we acknowledge that a moderate increase in temperature has, in certain circumstances, been known to induce thermal mechanotransductive responses.^152^

Non-thermal effects, on the other hand, arise as a consequence of (1) the acoustic radiation pressure that is imposed on a cell when the sound waves encounter it along their propagation pathway; (2) the normal and shear stresses experienced by a cell due to the fluid flow that is generated by the sound wave propagation (known as “acoustic streaming”) through the cell media; and (3) the extreme pressures and temperatures that the cell is subject to during cavitation that typically accompanies low frequency ultrasonic excitation.^146^

The acoustic radiation pressure is a steady, time-averaged normal force that is imparted on a cell as a consequence of its presence in an acoustic field. More specifically, it consists of the transfer of momentum from the sound wave to the cell when the propagating wave encounters it. The magnitude of the acoustic radiation pressure depends on the sound wave intensity, the cell dimension, the wave characteristics (wavelength, sound speed through the medium), and an acoustic contrast factor, which accounts for the relative densities and compressibilities between the medium and the cell. For the most part, cells, being of a dimension much smaller than typical sound wavelengths, at least in the low frequency ultrasound range below 1 MHz, tend to either be forced to translate under the influence of the acoustic radiation pressure in what is known as “acoustophoresis.”^154^ In a standing wave field, which typically arises due to reflections and hence superpositioning of waves along the confines of the cell chamber, the cells then end up being trapped at the nodes or antinodes of the standing wave.^155^ At high intensities, though, it is nevertheless possible for cells to fragment under the extreme mechanical stresses exerted on them by the acoustic radiation pressure.^156^

Acoustic streaming, on the other hand, comprises the flow, or momentum flux, that arises from pressure and velocity fluctuations that are generated in the liquid media due to viscous dissipation of the energy of the sound wave upon its attenuation as it propagates and is absorbed within the liquid. Again, at low ultrasonic frequencies, acoustic streaming mainly results in the translation of the cells along flow streamlines as a consequence of the viscous drag imposed on the cells. Other effects of either or both acoustic radiation pressure and acoustic streaming on cells can typically arise at much higher frequencies when the sound wavelength becomes comparable to the cell dimension, which we discuss in Sec. IV C.

**FIG. 7.**
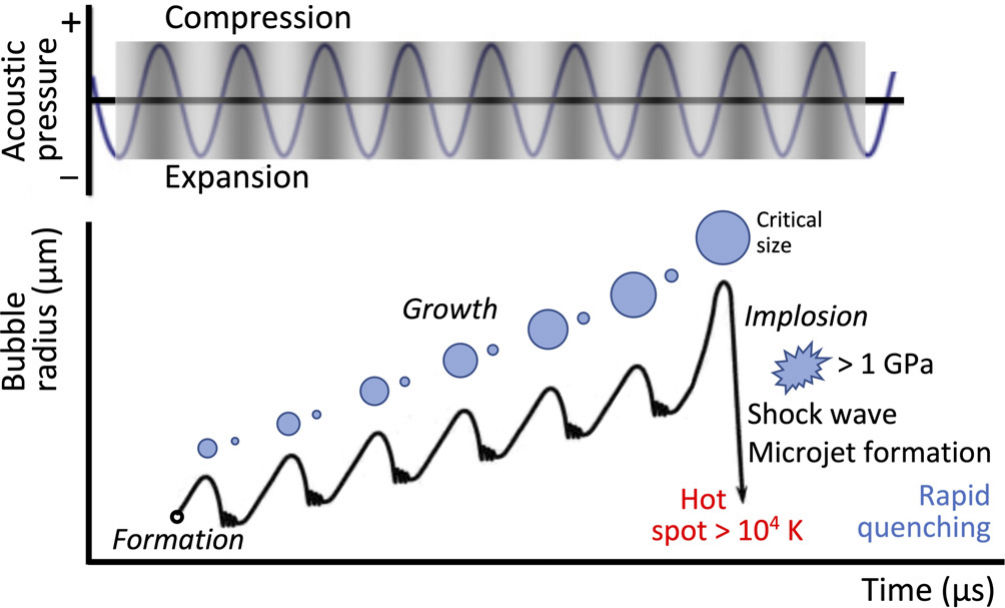
Acoustic cavitation. Schematic illustration depicting the formation, growth, and implosion of cavitation bubbles under low frequency ultrasonic excitation. Rezk *et al.*, Adv. Sci. **8**, 2001983 (2021); licensed under a Creative Commons Attribution (CC BY) license.

Among these effects, however, cavitation—the rapid growth, oscillation, and implosion of a bubble or an ensemble of bubbles beyond a critical sound wave intensity (Fig. 7)—by far, constitutes the central feature of low frequency ultrasonic mechanostimulation. Not only does the violent event associated with the rapid collapse of the bubble(s) lead to the formation of shock waves^157^ and high velocity microjets that induce intense local shear within the vicinity,^158^ extreme pressures ( >1 GPa), and temperatures ( $>10^{4}$ K) are also generated which can be extremely detrimental to cells.^147,159,160^ Additionally, cells are known to suffer from post-cavitation DNA damage when exposed to the free radicals that are typically produced during cavitation that lead to the production of reactive oxygen species (ROS).^161^

If moderate cell viabilities can be maintained at lower sound intensities, particularly at the higher end of the low frequency ultrasound range, whatever sufficient deformation experienced by the cell due to any of the aforementioned effects can potentially give rise to various mechanically activated cell signaling pathways.^149,150^ While the exact mechanism by which such mechanotransduction arises is as yet unclear, the deformation of the cell membrane likely plays a key role in the mechanosensory process. Pore formation in the cell membrane (sonoporation), which is commonly observed in low frequency ultrasonic excitation particularly as a consequence of cavitation,^162,163^ and which constitutes the dominant mechanism that underpins ultrasound-induced intracellular delivery,^164^ is expected to alter the membrane curvature, which, in turn, can activate SACs, such as the TRP and piezo channels.^165–167^ Additionally, transmembrane receptors such as integrins ( α and β) in many cells (including fibroblasts, osteoblasts, and chondrocytes)^168–170^ and FAs^169,171^ have been reported to alter with low frequency acoustic stimulation.^89,172^

These effects, and, in some cases, even cavitation-induced ROS production,^162,173,174^ have been claimed to alter intracellular Ca^2+^ concentration, leading to cytoskeletal reorganization both with or without FAK activation,^175,176^ while integrin activation is suggested to trigger the PI3K/Akt (phosphatidylinositol-3-kinase/serine–threonine kinase) pathway.^169^ In addition, low frequency ultrasonic mechanostimulation has also been reported to regulate the ERK/MAPK,^168,170,177^ nuclear factor kappa B (NF- κB),^178,179^ Rho–ROCK/ERK,^177^ and Wnt/ β-catenin pathways,^91,180^ in addition to calcineurin–NFAT (nuclear factor for activated T cells) activation^179^ and YAP nuclear translocation in MSCs.^181^ In ECs, regulation of expression of vascular endothelial growth factor (VEGF) and interleukin-8 (IL-8) has been reported.^182,183^

The translation of these signaling pathways can be observed in various downstream effects and cell fate in response to the low frequency ultrasonic mechanostimulation, such as MSC proliferation and upregulation of many cyclins,^180,184^ along with improvement in cell migration and an increase in adhesion molecules such as CXCR4, integrin‐1 β, and CCR2.^172,185^ Additionally, upregulation in Nanog expression has also been observed with such stimuli to thereby maintain the stemness of mouse primary osteoblasts and mesenchymal stromal stem cells.^186^ Depending on the culture conditions (e.g., the presence of biochemicals), low frequency ultrasound stimulation of multipotent stem cells can promote osteogenesis,^187^ chondrogenesis,^188^ neurogenesis,^189,190^ and adipogenesis.^191^ The commitment to osteogenic lineage in MSCs in the absence of chemical differentiation factors under low frequency ultrasound (stimulated for 28 days) has also been reported.^167,192–195^ In ECs, low frequency ultrasonic stimulation has been shown to improve proliferation and adhesion,^183,196^ in addition to stimulating angiogenesis^182,183^ to promote tube formation in human umbilical vein endothelial cells (HUVECs).^197^

A drawback, nevertheless, of low-frequency ultrasonic mechanostimulation is the long times (typically days) that are commonly required for the cells to be exposed to the irradiation to evoke more complex cellular responses.^90,192^ Such prolonged exposure often evokes deleterious effects on the cells, which include a considerable reduction in cell viability as well as a greater chance of contamination.

### C. High frequency ultrasonic bulk, surface, and hybrid waves (1 MHz–1 GHz)

High frequency ultrasonic waves have yielded completely distinct and often nonlinear phenomena when coupled into fluids and materials,^145^ stemming from the smaller sound wavelengths that approach the characteristic system length scale—in this case, that of the cell dimension or the confinement they are present within. Moreover, cavitation, which tends to be the dominant mechanism responsible for a host of phenomena driven by low frequency ultrasonic excitation, such as membrane poration, which we have seen in Sec. IV B to trigger a variety of mechanotransduction cascades, becomes increasingly non-existent at higher frequencies since the threshold sound intensity required to generate cavitation scales as the inverse of frequency.^145,198^ In addition to therefore involving an alternative mechanosensory mechanism for the excitation of mechanotransductive responses in the cell, the absence of cavitation allows for considerably greater retention of cell viabilities (typically >90% for high frequency ultrasound compared to around 60%–80% for low frequency ultrasound has generally been reported).^199^

Moreover, only short-duration exposure (typically up to 10 min per day for several days) of 10 MHz order surface (SAW) and hybrid (SRBW) wave excitation, for example, was generally required to evoke similar cell mechanoresponses^84,92^ that would have otherwise necessitated considerably longer exposure times with low frequency ultrasound (typically continuous excitation over several days). In these instances, perturbation of the cell membrane as a result of the SAW or SRBW excitation was reported to lead to activation of the SACs, and, in particular, the piezo channels,^84,200^ to drive an influx of extracellular Ca^2+^ into the cytoplasm. This, in turn, resulted in a transient decrease in the Ca^2+^ concentration in the immediate vicinity between the intercellular junctions to faciliate rearrangement of the AJ (e.g., cadherin) structure.^87^ Such cytoskeletal rearrangement is then responsible for triggering a series of downstream signaling cascades. In MSCs, MAPK signaling, for example, was seen to be activated when chemical factors were absent, whereas the Wnt/ β-catenin pathway appeared to dominate in the presence of chemical cues.^84^ Concurrently, the transient increase in the Ca^2+^ concentration in the cytoplasm promotes storage of intracellular Ca^2+^ in the endoplasmic reticulum (ER) by activating the sarcoplasmic reticulum Ca^2+^-ATPase (SERCA) pump,^92^ which subsequently led to activation of IP3R (inositol 1,4,5-trisphosphate receptor) and RyR to trigger signaling by the secondary second messenger cyclic adenosine monophosphate (cAMP). Downstream effects that transpire then include Rho–ROCK, ERK1/2 MAPK, and BMP2 signaling, as well as activation of the ESCRT (endosomal sorting complexes required for transport) and Epac1 (cAMP-activated guanine nucleotide exchange protein 1)–Rap1 (Ras-related protein 1) pathways to drive a variety of cellular activities depending on the type of cell that was stimulated^84,92^ (to be discussed in Sec. V).

A few studies have also found similar increases in intracellular Ca^2+^ in various cells, including HUVECs,^201^ Chinese hamster ovary (CHO) cells, and both invasive and noninvasive cancer cells,^202^ under high-frequency bulk waves, albeit with longer exposure durations over several hours. This was attributed to SACs gating, such as that of piezo channels, when both CHO and epithelial human breast cancer (MDA-MB-231) cells were stimulated at approximately 40–50 MHz at intensities of approximately 90 W/cm^2^.^202^ The involvement of TRP channels in cancer cells^203^ or connexin-43 in MSCs^88^ has also been reported. In the latter, the opening of connexin-43 is accompanied by the release of adenosine triphosphate (ATP) into the extracellular space, which, in turn, results in the activation of phospholipase C followed by Ca^2+^ release from the ER.^88^ As with other mechanostimulation studies using different frequencies and types of waves, it is expected that a myriad of downstream signaling cascades can be activated, although most investigations involving high-frequency bulk wave excitation (with the exception of Ref. 88) have focused primarily on demonstrating their effect on cell proliferation (see, for example, Refs. 204 and 205) and therefore its potential utility, for example, for the development of cancer therapeutics.^206^

## V. APPLICATIONS OF ACOUSTICALLY DRIVEN MECHANOSTIMULATION

Given the diverse mechanoresponses of cells to mechanostimulation driven by acoustic excitation in Sec. IV, sound waves, particularly at ultrasonic frequencies, have been employed to modulate a range of cellular activities that include cell growth, adhesion, proliferation, migration, death, and lysis.^149,207–209^ Additionally, there is a large body of literature on acoustically mediated intracellular delivery, enabled primarily by poration of the membrane by the acoustic forcing (i.e., sonoporation) that arises predominantly through cavitation (see, for example, the reviews by Coussios and Roy,^210^ Sitta and Howard,^164,211^ and Rich *et al.*^212^) although poration-free acoustic membrane permeabilization methods for intracellular delivery using high frequency (>10 MHz) excitation that are equally as efficient but also facilitate significantly higher retention in cellular viability have also been recently reported.^199,213–216^

In this section, we discuss a number of exemplary applications where acoustically driven mechanostimulation of different cell types can be deployed. These have primarily been focused on applications in regenerative therapeutics given the considerable work that has been carried out to date on the application of mechanical forces to control *in vitro* cell behavior for tissue remodeling and regeneration,^217–219^ although we also dedicate a brief discussion at the end of this section to other applications. As the scope of the present work is limited to acoustic modulation of internal cellular processes, we have excluded applications of ultrasonic technology for the physical manipulation of cells, such as cell trapping and sorting, or rotation (e.g., for spheroid generation^220–222^), for which we refer the reader to the various reviews available on this subject (see, for example, Refs. 9, 220, 223, and 206).

### A. Cell proliferation

Given their multipotency, trophicity, immunosuppressive properties, and capacity for self-renewal, stem cells, and, in particular, MSCs, are attractive candidates for regenerative therapy. Depending on the targeted disease/application, the required stem cell dose can range from $0.2\times 10^{6}$ to $1.2\times 10^{9}$ cells, and often involves multiple treatment courses.^224^ As such, the isolation and processing of these required numbers of cells constitutes the current bottleneck in practical stem cell therapies. Effective techniques for high-throughput *in vitro* stem cell expansion that can also address challenges associated with donor-related heterogeneity are therefore required.^225^ As constituents within the expansion media itself can potentially alter the characteristics of the expanded cells,^226^ efforts have predominantly been focused on developing mechanical intervention methods as an alternative strategy to chemically augmented cell proliferation.

Acoustic stimulation ranging from tens of Hz to approximately 150 MHz, as short as 5 min/day for one day through to continuous treatment over 12 days, has been noted to enhance the proliferation rate in various types of cells, irrespective of species or tissue source (Table II). While the propensity for cells to proliferate typically increases with vibration-induced stimuli, it is difficult to generally conclude how cell proliferation is affected by specific system parameters (frequency, intensity or exposure duration) given that these studies were conducted on widely varying cell types and conditions, often with markedly distinct trends observed. For example, while the proliferation rate of primary ECs was seen to be generally enhanced irrespective of applied frequency or intensity, that of nonmalignant cells tended to increase under low intensities on the order of 10 W/cm^2^, while malignant cells appeared to be more responsive to high intensity stimulation on the order 100–1000 W/cm^2^ (Table II). As such, cell type is likely to be a key determinant in the cell proliferation response to acoustic stimulation.

**TABLE II.** Summary of studies demonstrating modulation of cell proliferation through acoustic mechanostimuli.

Cell	Frequency	Intensity	Exposure time	Summary
Fibroblasts^232^	45 kHz	15 and 50 mW/cm^2^	5 min	30% and 43% increase in DNA content within 18 h of post-exposure incubation, as an indicator of the increase in cell proliferation
Osteoblasts^232^	45 kHz	5 and 30 mW/cm^2^	5 min	32% and 35% increase in DNA content within 18 h of post-exposure incubation, as an indicator of the increase in cell proliferation
Human amnion-derived mesenchymal stem cells (hAD-MSC)^94^	250 kHz	30 mW/cm^2^	30 min/day for a day	Increase in DNA content, as an indicator of increased cell proliferation
Mouse myoblasts (C2C12)^228^	0.5, 1, 3, 5 MHz	250–1000 mW/cm^2^	24 h	Improved proliferation
Bovine aortic endothelial cells^229^	0.5, 1, 3.5, and 5 MHz	1.2 W/cm^2^	5, 15, and 30 min/day for 3 days	Improved proliferation was detected within 24 h of post-exposure incubation although the proliferation rate was dependent on intensity
Human cardiac microvascular endothelial cells (hcMEC)^227^	0.51, 0.994, and 4.36 MHz	3, 25, 50, and 600 W/cm^2^	10 min (low intensity), 20 min (high intensity)	Enhanced proliferation at all frequencies irrespective of intensity
Fibroblasts^232^	1 MHz	0.7 and 1.0 W/cm^2^	5 min	47% and 37% increase in DNA content within 18 h of post-exposure incubation, as an indicator of the increase in cell proliferation
Osteoblasts^232^	1 MHz	0.7 and 1.0 W/cm^2^	5 min	34% and 52% increase in DNA content within 18 h of post-exposure incubation, as an indicator of the increase in cell proliferation
Rat Schwann cells^234^	1 MHz	27.25 W/cm^2^	10 min/day for 5 days	Improved proliferation
Rat pheochromocytoma cells (PC-12)^235^	1.48 MHz	0.1 MPa	5, 10, 20, and 30 min/day for 3 days	138%–166% increase in proliferation
Human nucleus pulposus cells (HNPSV-1)^236^	1.5 MHz	7.5, 15, 30, 60, and 120 mW/cm^2^	20 min/day for 5 or 12 days	Improved cell proliferation
Human bone marrow derived mesenchymal stem cells (hBMD-MSC)^233^	1.5 MHz	30, 40, 50, 60, and 80 mW/cm^2^	5–20 min/day for 4 days	Increase in proliferation with 50 and 60 mW/cm^2^ with at least 5 min incubation per day
Mouse myoblasts (C2C12)^237^	1.5 MHz	30 mW/cm^2^	20 min/day for 8 days	Increased proliferation rate and cell number
Primary skin fibroblasts^168^	1.5 MHz	30 mW/cm^2^	6–11 min/day for 7 days	Improved proliferation with 11 min stimulation
Rabbit knee synovial membrane cells (HIG-82)^238^	3 MHz	30 mW/cm^2^	15 min	No effect on cell proliferation
Mouse neuroblastoma cells (Neuro2A), Human colon adenocarcinoma cells (HT29)^227^	0.51, 0.994, and 4.36 MHz	3, 25, 50, and 600 W/cm^2^	10 min (low intensity), 20 min (high intensity)	Positive response to 4.36 MHz, 600 W/cm^2^ but decreased proliferation at 50 W/cm^2^; in both cell lines, the proliferation rate decreased after 120 h
Madin–Darby canine kidney epithelial cells (MDCK)^227^	0.51, 0.994, and 4.36 MHz	3, 25, and 50 W/cm^2^	10 min (low intensity), 20 min (high intensity)	Unresponsive to 4.36 MHz, 50 W/cm^2^ but improved proliferation rate at 4.36 MHz, 25 W/cm^2^; highest proliferation rate at 3 W/cm^2^
Human mesenchymal stem cells (hMSC)^184^	5 MHz	20 mW/cm^2^	5 min/day for 14 days	Increase in proliferation
Human monocytes cell line (U-937)^239^	48.8 MHz	467 mW	48 h	36% increase in cell proliferation
Madin–Darby canine kidney cells (MDCK-II), Human osteosarcoma sarcoma osteogenic cells (SaOs-2), human embryonic kidney cells (T-REx-293), Human monocytic tumor cells (U-937)^201^	100 MHz	80 mW/cm^2^	5 min–27 h	Increase in cell proliferation
Human osteosarcoma cells (SaOs-2)^240^	159 MHz	300 mW/cm^2^	5 h	Increase in proliferation

Moreover, the exact mechanisms by which cell proliferation is altered by acoustic stimulation are still not well understood. As heat is generated due to viscous absorption of the sound wave as it is transmitted through the media, most studies involving acoustic mechanostimulation incorporate some attempt to control fluctuations in temperature, as these can trigger thermally dependent mechanotransductive processes.^227,228^ Even then, the various mechanisms associated with non-thermal mechanical effects remain complex. Given the ubiquitous involvement of Ca^2+^ upregulation and Rho–ROCK activation across all forms of acoustic stimulation, as discussed in Sec. IV, and the corresponding changes to the cytoskeletal structure and FAs,^168,229–231^ it is probable that Ca^2+^-induced signaling triggers upregulation of YAP that, in turn, enhances actin nucleation and stability, cytokinesis, and cell cycle progression^181^ along with improved expression of growth factor receptors and cyclins, such as cyclin D1, E1, A2, and B1.^94,184,232^ Other possible signals triggered by acoustic stimulation that have also been implicated in mediating cell proliferation include the ERK1/2, Akt serine/threonine kinase,^94,233^ and glycogen synthase kinase-3 β (GSK- 3β)/ 3β-catenin^234^ pathways. However, we caution that careful consideration of the extent of stimulation applied is necessary given that prolonged exposure is known to inhibit cell growth or to induce apoptosis.^233^

### B. Cell migration

Cell migration is a complex process involving changes to tension in the cell membrane in addition to rearrangement of the cytoskeletal structure^241^ that is critical to various cellular activities, including homeostasis, morphogenesis, immune cell trafficking, cancer metastasis, and wound healing.^242^ In stem cells, mobilization (migration and homing of cells) to defective regions is critical for successful regeneration.^243^ In the bid to improve regenerative therapy, various mechanical loads, including acoustic forcing at frequencies ranging from a few Hz to MHz with intensities between 20 and 250 mW/cm^2^ (Table III) together with the addition of biochemical stimuli, have been explored to regulate cell migration.^242–245^ Based on the available data, higher frequencies, particularly that at MHz order, appeared to facilitate more cell migration over lower frequencies. A lone study nevertheless found that 20 kHz acoustic stimulation, in contrast, reduced migration of smooth muscle cells, with the baseline migration only being restored after 24 h of post-exposure incubation.^246^

**TABLE III.** Summary of studies demonstrating modulation of cell migration through acoustic mechanostimuli.

Cell	Frequency	Intensity	Exposure time	Summary
Immortalized mouse olfactory ensheathing cells (OECs)^253^	20, 60, and 80 Hz	2.173*g*	“Long durations” (not specified; 6, 12, and 24 h in prior study)	60 Hz stimulation produced larger spheroids (70-fold), indicating better migration
Human lung fibroblasts (LL24), Mouse fibroblasts (L929)^139^	100 Hz–1.6 kHz	0.2 W	5 min	Observation over 4 h showed frequency-dependent improvement in cell migration and migration distance; both cells showed increased migration (approximately 10%) at 100 Hz, although this decreased unsteadily with increasing frequency
Mouse fibroblasts (L929)^247^	11.2 kHz	2, 4 V	24 h	Vibration in the orthogonal direction to the gap of the scratch in the wound healing assay: twofold increase in migration with 2 V vibration but decrease in vibration at 4 V; vibration parallel to the gap: both 2 and 4 V vibration led to reduced migration rate
Bovine aortic smooth muscle cell (SMC)^246^	20 kHz	1.5 W	15 s	Observations at 0.2, 2, and 24 h post-exposure incubation showed 2.4-fold decrease in migration after sonication immediately following stimulation; this recovered slowly with post-exposure incubation but still remained at levels below that of untreated cells
Odontoblast-like cells (MDPC-23)^248^	45 kHz	25 mW/cm^2^	30 min	Cell proliferation and rate of wound healing significantly reduced in the presence of cell proliferation inhibitor mitomycin C (MMC), although not fully quelled; no effect on cell migration
Mouse calvarial derived osteoblasts (MC3T3-E1)^249^	45 kHz (continuous), 1 MHz (pulsed)	25 mW/cm^2^ (45 kHz), 250 mW/cm^2^ (1 MHz)	30 min	Both frequencies promoted migration in the presence of MMC (which has no significant effect of migration), although MHz stimulation displayed greater increase
Human epidermal keratinocyte cells (HaCaT)^252^	0.5 MHz	0.3 MPa	1 min	Migration improved by approximately 50%
Immortalized human chondrocytes (C-28/I2)^254^	1 MHz	30 mW/cm^2^	24, 48, and 72 h	Increased migration rate with or without the presence of cytokines (IL-1b)
Mouse calvarial derived osteoblasts (MC3T3-E1), Mouse osteosarcoma cells (LM8), Human osteosarcoma cells (SaOs-2), Human renal cancer cells (786-O), Human prostate cancer cells (PC-3), Human lung cancer cells (A549)^255^	1.5 MHz	30 mW/cm^2^	20 min/day for 3 days	Migration of MC3T3-E1 increased by 8.7% and 9.4% at 6 and 12 h, respectively, whereas the other cells were unaffected
Human periodontal ligament stem cells (PDLSC)^256^	1.5 MHz	30, 60, and 90 mW/cm^2^	30 min	Migration rate improved after 24 h post-exposure incubation
Normal human urothelial cells (NHU)^257^	1.5 MHz	30 mW/cm^2^	20 min/day for 2 days	No effect on migration
Rat bone marrow derived stem cells^258^	3 MHz	20, 30, 40, and 50 mW/cm^2^	20 min/day for 10 days	Increased migration rate, particularly at 50 mW/cm^2^
Mouse embryonic fibroblasts (NIH-3T3)^230^	14 MHz	59.3 mW cm^2^	4–8 h	Migration speed increased by 42% after 4 h post-exposure incubation although significant retraction of cells observed at higher intensities
Madin–Darby canine kidney cells (MDCK-II)^201^	100 MHz	80 mW cm^2^	27 h	Increased migration rate by 135% in addition to a significant increase in the rate of cell growth
Human osteosarcoma cells (SaOs-2)^240^	159 MHz	2–4 mW	48 h	Migration rate increased as a function of intensity with no preference in direction

In addition to promoting migration, continuous acoustic stimuli have been reported to orientate cells along the direction of the acoustic wave propagation, at least in fibroblasts,^201,230,247^ although this observation was not always universal. For example, while osteosarcoma exposed to 159 MHz at an intensity of 300 mW/cm^2^ displayed improved migration, there was no preference for its orientation.^240^

The fundamental mechanisms that govern such random or directed collective migration under acoustic stimulation are unclear. A complication is that migration effects are typically accompanied by cell proliferation and is often unclear if the improvement in migration is due to the increased proliferation rate or the actual movement of the cell. At least in one study, it was seen that acoustic stimulation at 45 kHz and 1.5 MHz frequencies under intensities between 25 and 250 mW/cm^2^ led to reductions in the migration rate of odontoblast and osteoblast cells in the presence of mitomycin C (MMC)—a cell proliferation inhibitor, suggesting that the proliferative capacity of the cells under acoustic stimuli dominates their propensity to migrate.^248,249^

Similarly, little is understood about the mechanotransductive processes that govern the migration of cells under acoustic stimuli. What is known to date is the involvement of the ECM, FA and cytoskeletal structures.^250^ In particular, acoustic stimuli have been reported to activate FA proteins, vinculin and tubulin, that, in turn, give rise to rearrangement of the cytoskeletal network.^246,251^ Additionally, both FAK and Ras-related protein 5 (Rab5) have been found to regulate cell migration driven by the acoustic stimulation,^86^ whereas mechanosensitive molecules such as Ca^2+^ and ROS (reactive oxygen species) are known to regulate the activation of PI3K/Akt and JNK (c-Jun N-terminal kinase) pathways, in addition to Rac1 (Rac Family Small GTPase 1) signaling,^201,251,252^ which increase the formation of lamellipodia and membrane ruffling to result in increased cell spreading and the formation of actin filopodia to drive cellular migration.^139^

### C. Angiogenesis

Acoustically induced mechanical loading has been shown to influence angiogenesis^183,196,229,259^—the *in situ* formation of new vasculature through a complex process that involves the proliferation, migration, and differentiation of vascular ECs—to thereby facilitate tissue regeneration and wound healing. Exposure of these cells to 0.5–1 MHz acoustic stimulation at intensities between 0.3 and 2.2 W/cm^2^ for 5–30 min, for example, displayed enhanced propensity for proliferation and migration, that, in turn, promoted sprouting of new capillaries.^183,196,229^ It has been claimed that the microstreaming from cavitation-induced microbubbles generated as a consequence of acoustic stimulation at low ultrasonic frequencies (1 MHz) also leads to improved cell proliferation and migration, both of which are crucial for angiogenesis, although it is unclear if this was a direct consequence of either the vibrational stimuli on the cells and the membrane deformation and corresponding SAC activation that is induced, or an indirect consequence of the shear stress imposed by the microstreaming.^150^

In any case, the exact fundamental mechanism by which angiogenesis is triggered by mechanostimuli, in general, is not well understood, although the role of mechanosensitive molecules, such as Ca^2+^, nitric oxide (NO) integrins, FAKs, and caveolin, have been implicated in the process.^260^ In particular, acoustic stimulation has been shown to increase intracellular Ca^2+^ and NO to effect cytoskeletal rearrangement in ECs.^261,262^ It is then possible that these induce nuclear translocation of YAP and activation of the Akt signaling cascade to result in improved vascular endothelial growth factor (VEGF) expression that accompanies angiogenesis.^93,259^ However, due care has to be taken when exposing the ECs to these low frequency ultrasonic waves to avoid inducing apoptosis in the cells.

### D. Differentiation

Mechanical stimuli involving laminar shear or cyclic stretching/pressure have long been adopted to guide the differentiation fate of stem and progenitor cells, particularly toward osteogenic lineages,^126,263,264^ although these have typically been administered in the presence of at least one biochemical stimulant. Bulk acoustic stimuli at frequencies ranging from Hz to MHz have also been used to instigate various differentiation fates, particularly osteogenesis, in stem cell or progenitor cells, albeit typically in the presence of biochemical factors as well (Table IV). The focus of the majority of these studies has, nevertheless, been primarily rooted in the demonstration of the mechanostimuli for stem cell differentiation, rather than explicating the fundamental mechanisms by which the acoustic stimulation drives the process. That the mechanostimuli have been applied in the presence of the biochemical differentiation factors, however, has led to the prevalent thought that the observed changes in the protein profile can primarily be attributed to the chemical rather than the acoustic stimuli.^265^

**TABLE IV.** Summary of studies demonstrating acoustically induced osteogenesis in various stem and progenitor cells.

Cell	Frequency	Intensity/acceleration/amplitude	Culture condition	Exposure time	Summary
Human bone marrow derived mesenchymal stem cells (hBMD-MSC)^292^	30, 400, and 800 Hz	0.3*g*	Osteogenic media	30 min/day for 14 days	Improved osteogenesis with 800 Hz stimulation
Human mesenchymal stem cells (hMSC)^193^	50 Hz–1 kHz	10 and 20 V	Basal media	7 days continuous stimulation	Moderate improvement of RUNX2 expression (0.4-fold) with 1 kHz stimulation
Rat bone marrow derived mesenchymal stem cells (rBMD-MSC)^293^	60 Hz	0.3*g*	Osteogenic media	1 h/day on days 0, 1, 2, 4, 5, and 6	Observations at day 14 showed improved osteogenesis
Human mesenchymal stem cells (hMSC)^195^	500 Hz–10 kHz	3.6 and 20*g*	Basal media	21 days of continuous stimulation with nanotopography	Synergistic action of nanovibrations and square-shaped nanotopography with viteronectin improved osteogenesis based on the frequency of nanovibration
Co-culture (human bone marrow hematopoietic cells, osteoclast precursors, mesenchymal stromal cells, osteoprogenitors, osteoblasts, and osteocytes)^266^	1 kHz	44.4 nm	Basal media	7 days continuous stimulation	Both 2D and 3D culture (collagen matrix with collagen 2–5 mg/ml) showed improved osteogenesis and inhibited osteoclastiogenesis in hBMD-MSC/hBMHC
Skeltol stem cells selected from bone marrow stroma^192^	1 kHz	30 and 90 nm	Basal media with collagen hydrogel (2–5 mg/ml) of stiffness 26 Pa	9 days continuous stimulation	Improved osteogenesis
Human bone marrow derived mesenchymal stem cells (hBMD-MSC)^167^	1 kHz	20 nm, 80 V	Basal media with collagen-based hydrogel scaffolds (2.05 mg/ml)	21 days continuous stimulation	Improved osteogenesis
Rat bone marrow derived mesenchymal stem cells (rBMD-MSC)^294^	0.6 MHz	30 mW/cm^2^	Osteogenic media	20 min/day for 13 days	Improved osteogenesis
Rat bone marrow derived mesenchymal stem cells (rBMD-MSC)^295^	1 MHz	400 mW/cm^2^	Osteogenic media with β-tricalcium phosphate and poly(L-lactic acid) scaffold	20 min/day for 16 days	Improved osteogenesis
Human mesenchymal stem cells (hMSC)^296^	1 MHz	200 mW/cm^2^	Osteogenic media with arginin-glycine-aspartate (RGD)-grafted oxidized sodium alginate/N-succinyl chitosan	10 min/day for 28 days	Along with RGD, mechanostimulation improved osteogenesis
Human adipose derived mesenchymal stem cells (hAD-MSC)^297^	1 MHz	30 mW/cm^2^	Osteogenic media under microgravity	20 min/day for 12 days	Under microgravity, osteogenesis reduced significantly but was restored under mechanostimulation
Human alveolar bone-derived mesenchymal stem cells (hABD-MSC)^298^	1 MHz	50 mW/cm^2^	Osteogenic media	10 min/day for 3 weeks	Improved osteogenesis
Human mesenchymal stem cells (hMSC)^177^	1.5 MHz	30 mW/cm^2^	Osteogenic media with magnesium-doped hydroxyapatite/collagen hybrid scaffold	20 min/day for 7 and 14 days	Improved osteogenesis
Human bone marrow derived mesenchymal stem cells (hBMD-MSC)^299^	1.5 MHz	150 mW/cm^2^	Osteogenic media with arginin-glycine-aspartate-serene and nanochrystaline hydroxyapatite	5 min/day for 3 weeks	Improved osteogenesis with synergistic effect with 3D printed bioactive scaffolds
Human mesenchymal stem cells (hMSCs)^265^	1.5 MHz	30 mW/cm^2^	Basal and osteogenic media	20 min/day for five consecutive days per week, up to 3 weeks	Improved osteogenesis in osteogenic media
Mouse preadipocyte cells (3T3-L1), Mouse mesenchymal stem cells [ST2 and 10 T(1/2)], Subclone 4 preosteoblasts (MC3T3-E1), Osteoblasts^300^	1.5 MHz	30 mW/cm^2^	Adipogenic and osteogenic media	20 min/day for 15 days	Adipogenesis was suppressed in preadipocytes and MSCs upon stimulation; osteogenesis was improved in MSCs
Rat bone marrow derived stromal cells^301^	1.5 MHz	30 mW/cm^2^	Basal media with ascorbic acid as osteogenic stimulant	20 min single treatment	Observation at 0.5, 1, 3, 6, and 12 h post-exposure showed improved osteogenesis
Human mandibular fracture haematoma-derived cells (hMHCs)	1.5 MHz	30 mW/cm^2^	Osteogenic media	20 min/day for 20 days	Improved osteogenesis
Human adipose derived stem cells (hAD-SC)^302^	2 MHz	20 and 30 mW/cm^2^	Osteogenic media	30 min/day for 21 days	Improved osteogenesis with increase in heat shock protein-70 and heat shock protein-90 expression and BMP signaling pathway activation
Neonatal mice osteoblasts^303^	3 MHz	30 mW/cm^2^	Basal media	15 min/day for 10 days	Improved proliferation and mineralization; enhanced heat shock protein-90 expression
Human bone marrow derived mesenchymal stem cells (hBMD-MSC), Human adipose derived stem cells (hAD-SC), Human umbilical cord derived stem cells (hUCD-SC)^84^	10 MHz	2.5 W	Basal and osteogenic media	10 min/day for 5 days	Improved osteogenesis in both basal and osteogenic media; early osteogenic commitment with basal media while stimulated cells at ostegenic media showed similar patterns to that for control osteogenic media

An exception to the aforementioned studies is the work by Dalby and co-workers, who showed the possibility of directing MSCs toward osteogenic lineage using nanoscale-amplitude acoustic stimuli at kHz-order (50 Hz–10 kHz) frequencies without requiring any biochemical factors. In their studies, a modest improvement in the expression of the osteogenic marker RUNX2 (Runt-related transcription factor 2) was reported, particularly with 1 kHz mechanostimulation continuously for 7 days.^193^ This was ameliorated by co-culturing stem cells with osteoprogenitors, osteoblasts, and osteocytes, and also in collagen-based soft gel (0.26 Pa) scaffolds and on nanotopographically manipulated surfaces.^167,192,195,266^ The acoustic stimulation, delivered continuously for 27 days, resulted in upregulation of various osteogenic markers, such as RUNX2, bone morphogenic protein (BMP-2), osteocalcin (OCN), osteopontin (OPN), osteonectin (ONN), and osterix.

Of particular note is the finding in the aforementioned studies of little upregulation in osteogenic markers beyond 1 kHz, and hence the assertion that there is little benefit in operating at higher frequencies.^195^ Contrary to those findings, however, it has recently been shown that 10 MHz mechanostimulation involving SRBWs not just results in significant osteogenic marker upregulation in human MSCs from different tissue sources and donors without requiring chemical/biochemical stimuli, but that it also does so considerably earlier at day 3 following commencement of the stimulation, and with just 10 min of stimulation per day for 5 days.^84^ Despite such short stimulation periods, the cells are nevertheless observed to retain their long-term osteogenic potential, even after trypsinization and reseeding after 5 days. As such, there are appreciable advantages in using these higher frequencies since the early osteogenic commitment lowers the dedifferentiation risk in the stem cells.^267^ Moreover, the considerably shorter, non-continuous stimulation not only increases cell viability and proliferability but also offers a simpler and more cost-effective solution for clinical translation. In addition, the early, yet persistent, differentiation of SRBW-stimulated cells facilitates easier and more effective incorporation of committed cells into scaffolds for implantation^84,268^ or delivery direct to the injury site.^269^ This is because such early commitment allows the embedded or delivered cells to grow and adapt within the naturally evolving niche in real time, therefore circumventing the cumbersome need for designing and developing multicomponent scaffolds that mimic the natural niche during *ex vivo* or *in vitro* differentiation,^270–273^ in addition to facilitating the adaptation of the cells to different physiological needs for the development of personalized therapeutics.

The exact mechanostimulation-induced signaling cascade involved in osteogenesis is complex due to the involvement of multiple mechanosensors and pathways, and their interconnectivity. With mechanical loading, integrins,^59,172,274,275^ SACs such as TRP^124,167,276^ and piezo channels,^84,167,277^ cadherins, and connexins have been implicated in the process. The integrins trigger mechanotransductive processes that lead to activation of kinase pathways^169,278^ involving FAs such as paxillin and vinculin, whereas activation of SACs and the opening of connexin hemichannels result in increases in intracellular Ca^2+^ that initiates the MAPK pathway.^279,280^ On the other hand, mechanostimulation is also known to dissociate cadherins from auxiliary proteins such as β-catenin, whose concentration increase in the intracellular domains initiates the Wnt/ β-catenin pathway.^281,282^

**FIG. 8.**
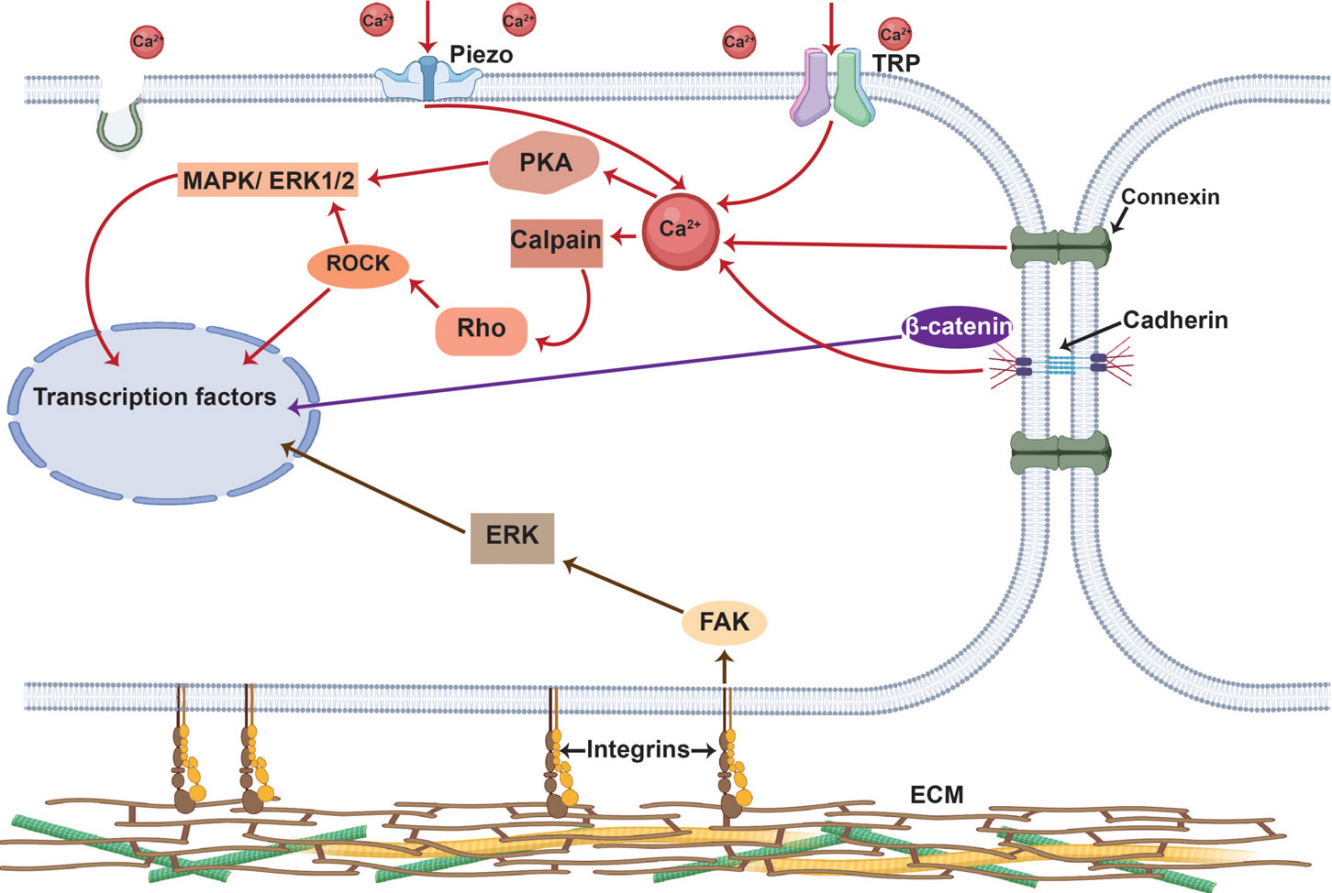
Schematic representation of the typical osteogenic pathways triggered by acoustically driven mechanostimulation of stem cells. The stimulation activates SACs, such as piezo and TRP channels, in addition to cell junctional proteins, such as connexins and cadherins, that facilitate influx of Ca^2+^. The change in the intracellular Ca^2+^ profile is responsible for initiating various Ca^2+^-signaling cascades (red arrows), such as Rho–ROCK (possibly through calpain) and PKA (cAMP-dependent protein kinase) signaling. While the former is responsible for regulating nuclear translocation of osteogenic factors such as TAZ and RUNX2, it can also initiate ERK/MAPK signaling such as PKA to instigate transcription factors, such as c-Fos, to induce osteogenesis. Concurrently, mechanostimulated integrins initiate the kinase pathway (brown arrows) through FAKs. The dissociation of β-catenin from cadherin, on the other hand, activates the Wnt canonical signaling pathway (purple arrows). ERK: extracellular signal-related kinase; FAK: focal adhesion kinase; MAPK: mitogen-activated protein kinase, Rho: Ras homologous protein; ROCK: Rho-associated protein kinase; TRP: transient receptor potential channel.

It was suggested that the low frequency (50 Hz–10 kHz) acoustic stimulation, in particular, directed activation of TRPV channels (which, being insensitive to membrane stretch,^30^ are gated through changes in temperature that affects the membrane phospholipids to drive changes in the membrane tension^26,27,283^) that, in turn, triggered the Wnt/ β-catenin pathway. In addition to such temperature-induced ion channel gating, it is also probable that the osteogenic differentiation in these studies could also be induced through the influence of the surface nanotopography of the substrates employed, or due to the presence of collagen, even though the scaffold used was considerably softer than those known to promote osteogenesis, since both the ECM and patterned substrates are known to participate in osteogenic differentiation through Wnt/ β-catenin and MAPK signaling.^284–287^ A different signaling transduction cascade, however, has been explicated for the SRBW mechanostimulation in the absence of any biochemicals, wherein the transient membrane aberrations that are induced by the excitation are suggested to activate piezo channels that, in turn, lead to Rho–ROCK or calpain activation to trigger bone morphogenetic protein-2 (BMP-2) production.^84^ Regardless, all of these pathways typically upregulate RUNX2, which commits the cells toward osteogenesis (Fig. 8).^288,289^

In addition to osteogenesis, acoustic stimuli have also been shown to direct stem cells toward other cellular fates, such as neurogenesis, adipogenesis, and chondrogenesis (Table V), although this always necessitated the inclusion of the specific biochemical differentiation factor. Interestingly, the incubation period between consecutive acoustic exposure at 90 Hz on embryonic MSCs was shown to have an inhibitory effect on adipogenesis,^290^ although another work at the same frequency on BMD-SCs, in the absence of such incubation periods between stimuli but again with the addition of adipogenic differentiation media, resulted in commitment to an adipogenic lineage.^191^ Most of these studies, however, do not examine the mechanotransduction pathways that are involved in the process, although it has been suggested that the ERK/MAPK pathway is activated in acoustically triggered neurogenesis in BMD-SCs.^189^ More broadly, it is likely from various studies that MAPK signaling can induce either osteogenesis, neurogenesis, chondrogenesis, or adipogenesis in BMD-SCs or chondrocytes depending on the biochemical factor that is added during the mechanostimulation.^141,191,290,291^

**TABLE V.** Summary of studies demonstrating acoustically induced stem cell differentiation toward non-osteogenic lineages.

Cell	Frequency	Intensity/acceleration	Culture condition	Exposure time	Summary
Rat bone marrow derived mesenchymal stem cells (rBMD-MSC)^191^	40 Hz	0.3*g*	Adipogenic media	15 min/day for 14 days	Improved adipogenesis
C3H10T1/2 embryonic MSCs^290^	90 Hz	0.7*g*	Adipogenic/multipotential media	Varying exposure ranging between 20 min/day to 6 h/day for 7–8 days	Two 20 min exposure with 1 h refractory period between each exposure suppressed adipogenesis; osteogenesis at day 8 improved by increasing refractory period to 3 h
Human bone marrow derived mesenchymal stem cells (hBMD-MSC)^189^	0.04 MHz	50 mW/cm^2^	Neuronal differentiation media	60 min/day for 3 and 7 days	Improved neurogenesis
Neural crest stem cells (NCSC) derived from human induced pluripotent stem cells (iPSC)^304^	1 MHz	500 mW/cm^2^	Neuronal differentiation media	10 min/day up to 4 days	Improved neuronal differentiation
Rat neural stem cells (rNSC)^305^	1 MHz	69.3 mW/cm^2^	Neuronal differentiation media	5 min/day for three consecutive days, 72 h from seeding	Observations at day 7 showed improved differentiation by modulating the Notch signaling pathway
Human mesenchymal stem cells (hMSC)^187^	1 MHz	200 mW/cm^2^	Chondrogenic/osteogenic media	20 min/day for 4 weeks	Improved differentiation to respective lineage based on media used
Rat bone marrow derived mesenchymal stem cells (rBMD-MSC)^190^	1 MHz	10, 30, 50, and 70 mW/cm^2^	Neuronal differentiation media	3 min/day for 3 day	Inhibited gliosis differentiation; did not promote neuronal differentiation
Murine embryonic stem cells^306^	1 MHz	21 and 147 mW/cm^2^	Embryoid body media containing cardiogenol	10 min/day from days 11 to 21	Increased late cardiac gene expression, indicating cardiomyocyte differentiation
C28/I2 human chondrocyte cells^291^	1.0 MHz	30 mW/cm^2^	Basal media	10 and 20 min	Upregulation in chondrogenic markers up to 1 day after stimulation
Human mesenchymal stem cells (hMSC)^307^	1.5 MHz	100 mW/cm^2^	Chondrogenic media with poly-(ethylene glycol) diacrylate scaffold	3 min for 3 weeks	Improved proliferation and chondrogenesis
Human mesenchymal stem cells (hMSC)^308^	1.5 MHz	30 mW/cm^2^	Chondrogenic media with poly-(ethylene glycol) diacrylate scaffold and microbubbles	3 min/day for 3 weeks	Improved chondrogenesis
Human gingival progenitor cells (HGPC)^309^	1.5 MHz	30 mW/cm^2^	Neuronal differentiation media	10 min/day for 3 days	Improved neurodifferentiation at day 4
Human embryonic stem cells (hESC)^310^	19.69 MHz	24, 31, and 39 V	Neuronal differentiation media	10 min/day for 12 days	Improved neurodifferenitation
Rat bone marrow derived cells (rBMDC)^311^	3 MHz	20, 30, 40, and 50 mW/cm^2^	Chondrogenic media	20 min/day for 10 days	Improved chondrogenesis
Rat bone marrow derived cells (rBMDC)^312^	3 MHz	20, 30, 40, and 50 mW/cm^2^	Chondrogenic media	20 min/day for 10 days	Improved chondrogenesis
Human chondrocytic cells (HCS)-2/8, Rat primary epiphyseal and articular cartilage cells, Ccn2-deficient chondrocytes^313^	3 MHz	60 mW/cm^2^	Basal media	20 min	Observations between 30 min and 5 h showed upregulation in chondrocyte differentiation markers

### E. Endothelial barrier modulation

The endothelial barrier, which partitions the intravascular and interstitial tissue compartments, plays a vital role in controlling the passage of solute and nutrient molecules in and out of organs. To enable such transport, its permeability is regulated by interactions between neighboring ECs that facilitate dynamic remodeling of the cytoskeletal structure through the modification of the associated junctional proteins. Given we have seen that cytoskeletal perturbation constitutes a dominant cellular response to various mechanostimuli, it then stands to reason that acoustic stimuli can potentially constitute a key driver of cytoskeletal remodeling and is hence a potent modulator of endothelial barrier function, which is crucial for tissure remodeling.

Indeed, 1 MHz-order bulk acoustic waves, either through direct mechanostimulation of ECs, or through the generation of cavitational microbubbles, have been exploited for enhancing the permeability of the endothelial barrier in both *in vitro* and *in vivo* conditions to facilitate transport of therapeutic molecules across the barrier.^162,314–316^ In either case, the acoustic stimuli, through the modification of the associated junctional proteins, were observed to drive influx of Ca^2+^ into the cell to induce the formation of actin stress fibers across the cell that, in turn, alters its contractility, leading to a change in the intracellular gap and hence the permeability of the barrier.^314–317^ We note the effect to be considerably larger in the case of bubble generation given their propensity to porate the cell membrane. In both cases, though, only slight increases in intracellular Ca^2+^ within the cell were observed, given that higher Ca^2+^ concentrations ( <10 μM) were associated with the inability for cells to recover from the imposed disturbance.^318^

More recently, 10 MHz SRBW mechanostimulation of endothelial monolayers has also been observed to facilitate modulation of endothelial barrier integrity: up to around fourfold increase in transendothelial electrical resistance (TEER), corresponding to a decrease in paracellular permeability by 5% that was sustained for over 4 h, was observed with just 8 min of SRBW excitation of ECs from different tissue sources (human umbilical vein, human saphenous vein, human coronary artery, and human aorta cells). Moreover, the effect was able to be sustained over longer durations through repeated treatments every 6 h.^87^

The same increase in intracellular Ca^2+^ influx into the cell as a result of transient membrane permeabilization and piezo channel activation under SRBW mechanostimulation in Sec. V D for stem cells, was observed to induce a local depreciation of Ca^2+^ near the junctions between the ECs, causing a reversible invagination of the immature zipper-like vascular endothelial (VE)-cadherin structure that forms the backbone of the AJs anchoring together the actin cytoskeletons of the ECs to form its barrier (termed the “sonochallenge” phase). This leads to the formation of mature linear VE-cadherin structures as a consequence of activation of the Ca^2+^-induced cAMP-activated guanine nucleotide exchange protein 1 (Epac1)–Ras-related protein 1 (Rap1) pathway (termed the “sonotransformation” phase). This, together with Ca^2+^ remodeling of the actin cytoskeletal structure, in which actin stress fibers across the cytosol were progressively replaced by circumferential actin bundles lining the cell periphery near its junctions, then resulted in a reduction in barrier permeability.^87^

The fourfold improvement in TEER with SRBW mechanostimulation is considerably higher than the 1.1–1.2-fold enhancement reported for chemical stimulation, therefore alluding to the SRBW as a potent means for improving endothelial barrier capacity in the development of *in vitro* models that more faithfully recapitulate *in vivo* barrier function—a current limitation of endothelial tissue models such as the blood–brain barrier, blood–retinal barrier or pulmonary air–liquid interface, and hence a bottleneck in the development and testing of therapeutic interventions and delivery strategies.^87^

**FIG. 9.**
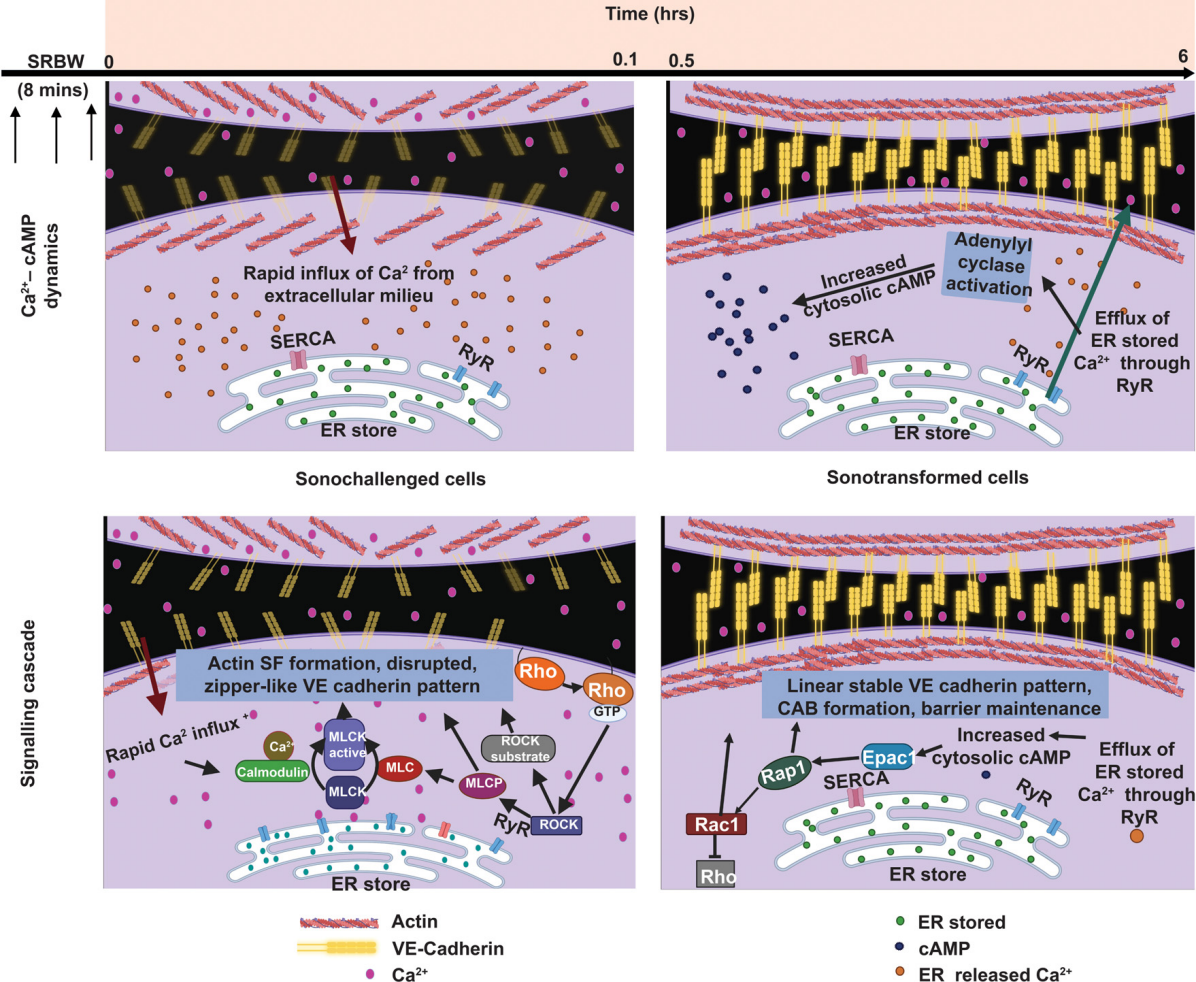
Calcium–cAMP signaling cascades induced by SRBW mechanostimulation. The immediate response of the SRBW-challenged cells is the sudden influx in intracellular Ca^2+^ as a consequence of membrane aberrations and piezo channel activation, which reduces Ca^2+^ levels in the immediate vicinity of the cells and drives transient invagination of VE-cadherin, while inducing Rho–ROCK signaling that causes actin stress fibers to be produced. Consequently, immature zipper-like VE-cadherin conformation ensues in the initial sonochallenge phase immediately following the 8 min SRBW excitation. As the cell subsequently relaxes, the increased intracellular Ca^2+^ level is ameliorated by their storage in the ER through SERCA, after which Ca^2+^ is slowly released back into the extracellular milieu through RyR activation. cAMP, formed by RyR activation, then initiates the Epac1–Rap1 pathway which further triggers the formation of circumferential actin bundles and mature VE-cadherin conformation in this subsequent, though simultaneous, sonotransformation phase in which the endothelial barrier integrity is enhanced. Reproduced with permission from Ambattu *et al.*, Biomaterials **292**, 121866 (2023). Copyright 2023 Elsevier Ltd.

The mechanotransduction process involved in the SRBW endothelial barrier modulation, illustrated in Fig. 9, clearly shows how acoustic stimulation is able to invoke the activation of multiple signaling cascades. Upon SRBW stimulation, permeabilization of the membrane and SAC activation leads to a rapid influx of Ca^2+^ into the cell. Such an increase in intracellular Ca^2+^ results in immediate Rho–ROCK activation given that Rho signaling simply involves phosphorylation/dephosphorylation, which triggers the formation of actin stress fibers in the initial sonochallenge phase. The disruption of the AJ structure into zipper-like patterns that ensues then leads to contraction of the cell and a corresponding increase in the intercellular gap that results in permeabilization of the endothelial barrier. This response is however transient, since the increase in intracellular Ca^2+^ also activates the SERCA pump to facilitate storage of surplus Ca^2+^ within the ER to maintain cytoplasmic Ca^2+^ homeostasis, as described in Sec. IV C. Upon relaxation of the SRBW excitation, the stored Ca^2+^ is subsequently released from the ER back into the extracellular milieu over time primarily through RyR activation. This is accompanied by activation of the Epac1–Rap1 pathway to result in formation of circumferential actin bundles and stable linear AJs in the sonotransformation phase, consequently resulting in an increase in the integrity of the endothelial barrier.^87^

### F. Exosome stimulation

In a manner akin to chemical intervention (e.g., subjecting cells to chemical additives such as calcium ionophores or calcium phosphate) or physical insults (e.g., inducing hypoxia or exposing cells to radiation), all of which serve to increase Ca^2+^ levels in the cell, the increase in influx of Ca^2+^ into cancer cells as a consequence of SRBW mechanostimulation due to aberrations generated on the cell membrane and piezo channel activation has been observed to activate various signaling pathways associated with cellular homeostasis. More specifically, the elevation of Ca^2+^ in the cytoplasmic matrix under the SRBW was observed to trigger sequestration of the accessory protein ALIX [ALG-2 (apoptosis-linked gene 2)-interacting protein X]^319^ at the site of the membrane aberrations to initiate exosome biogenesis,^92^ in which the membrane is remodeled to facilitate protein cargo sorting, plasma and nuclear envelope membrane repair, and the formation of multivesicular bodies (MVBs). In particular, membrane invagination resulted in the generation of intraluminal vesicles within these MVBs, which upon fusion with the plasma membrane, are released into the ECM as exosomes.^320,321^

**FIG. 10.**
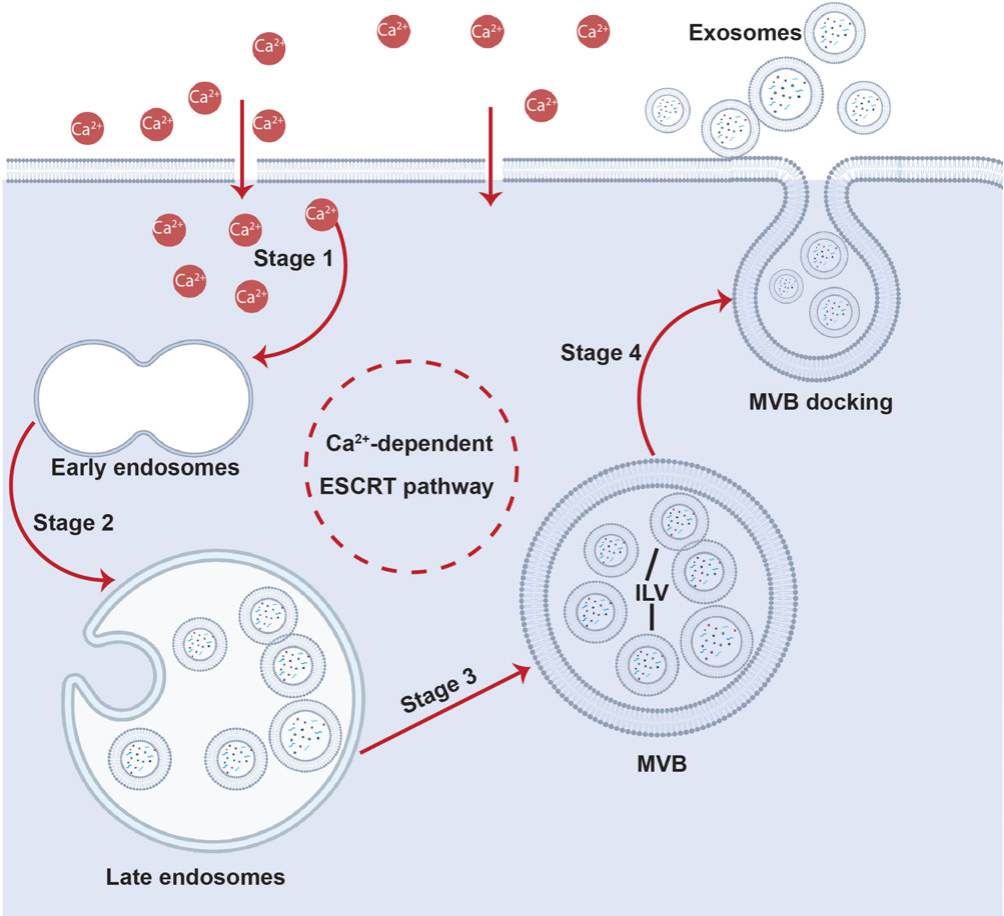
Proposed mechanism for SRBW-facilitated exosome production. The Ca^2+^ influx through membrane aberrations or piezo channel activation (stage 1) promotes the recruitment of ALIX—an accessory protein that makes up part of the ESCRT machinery—to the site along the membrane where the perturbation is introduced. The resultant ESCRT activation leads to the formation of early endosomes (stage 2) and their maturation into late endosomes (stage 3). These then transform into MVBs that subsequently dock onto the cell membrane (stage 4) to release the intraluminal vesicles (ILVs) within them as exosomes, all the while during which the aberrations in the cell membrane are being healed.^92^

As such, the SRBW-facilitated stimulation of exosome production can be viewed from the perspective of the cells' attempt to maintain homeostasis by repairing the transient membrane aberrations induced by the mechanical stimulation. In this process, the cell membrane acts as the mechanosensor with the intracellular Ca^2+^ as the mechanotransmission element and the ESCRT pathway as the mechanoresponse (Fig. 10). As with the endothelial and stem cell treatments in Secs. V D–V F, we note again that only short exposure to the SRBW (8 min) is sufficient to drive longer term effects of exosome release within a half hour post-exposure period. Given similarly high post-exposure cell viabilities (90+%), it is then possible to envisage cycling the SRBW irradiation to achieve tenfold enrichment in the exosome concentration to a homogeneous yield of around 1.7–2.1 fold/h, thereby constituting a facile but effective method for circumventing the perennial limitation of insufficient homogeneous exosome yield that constitutes the bottleneck in exosome diagnostics and therapeutics.^92^

### G. Applications beyond regenerative therapeutics

While the majority of applications involving acoustic mechanostimulation have predominantly been confined to those within the regenerative therapeutics space, there are also a number of other applications in which the utility of such stimulation has proven beneficial for the manipulation of a diverse range of cells, including immune cells [e.g., natural killer (NK) cells], neurons, oocytes, embryos, and sperm.^322–327^ Acoustically stimulating oocytes, for example, at 20 Hz and between 1 and 3 MHz with intensities of approximately 2 W/cm^2^, have been observed to maintain higher numbers of cells in the blastocyst (suggesting that the stimulation prevented cellular damage during oocyte activation), in addition to facilitating nuclear activation, which is dependent on the extracellular Ca^2+^ concentration,^328,329^ noting that 0.1–0.5 mM is optimal for oocyte activation.^323^ In fact, oocyte activation deficiency is the primary reason for total fertilization failure (TFF), with abnormal Ca^2+^ signaling being a key characteristic of the condition.^330^ Interestingly, similar increases in the rate at which embryos develop as a consequence of their activation have been observed when fused embryos are exposed to 1 MHz mechanostimulation at an intensity of 2 W/cm^2^ for just 30 s per day.^322^

Short duration (seconds) exposure of sperms to 19.28, 48.5, and 100 MHz SAWs with intensities ranging between 250 mW and 2 W, on the other hand, has been shown to influence their motility without compromising their structure or function.^325^ The influence of the acoustic stimulation on sperm motility was suggested to arise through the ability of the acoustic stimulation to mediate transport of Na^+^, H^+^, Ca^2+^, and/or HCO^3−^ into the cells by activating various ion channels, although the authors were compelled to attribute a lesser role to Ca^2+^ transport given their observations that sperm motility remained elevated even under Ca^2+^-free conditions. We note from their results though that the motility did appear to decrease from 34% to 19% in the absence of Ca^2+^, therefore implying that Ca^2+^ transport still has an effect on sperm motility, which would be consistent with prior studies reporting Ca^2+^ regulation of intracellular ATP and cAMP, both of which play a central role in sperm motility.^331,332^ While a large influx of Ca^2+^ is known to produce hypermotile sperm,^325^ we note Ca^2+^ influx to be a key driver in the activation of the cAMP/PKA pathway that regulates sperm motility.^325,331^ Given that it is the ratio of intracellular to extracellular Ca^2+^ that regulates the energy (ATP) required for sperms to swim,^333^ it is therefore likely that the magnitude of Ca^2+^ influx constitutes a key parameter in regulating sperm motility.

Acoustic stimulation has also been shown to offer a nondestructive, reversible, and adjustable means of manipulating neurons,^334^ giving rise to an entire field of sonogenetic neuromodulation for the regulation of the central, peripheral, or autonomic nervous system.^207,324,326,335–338^ Sciatic nerves and cortical neurons, excited by 100–800 MPa acoustic pressure at 0.8 and 27 MHz, for example, can be activated by acoustic stimulation,^339,340^ leading to an increase in their conduction velocity and action potential, thereby altering their excitability. Similar effects were noted in acoustically stimulated sciatic nerves at 2–7 MHz and 100–800 W/cm^2^.^341^ Meanwhile, a reduction in the action potential of sciatic nerves due to thermal effects was reported with acoustic stimulation at 0.661 and 1.986 MHz with an intensity of 44 W/cm^2^.^342^

Increased neural activity of cells in the superficial layer of salamander retina with 43 MHz bulk ultrasound has, in addition, been reported, although the effect failed to be reproduced in retinal ganglion cells.^343^ Although the precise mechanism by which neuron excitability is modulated through acoustic stimulation was not explicated in this study, it is possible that their observations can be attributed to piezo channel activation, which was reported in HEK293 cells exposed to bulk ultrasound excitation at similar frequencies,^202^ and since SACs such as piezo channels are endogenously expressed in neurons.^31^ Moreover, the excitation of mouse neurons under 500 kHz bulk mechanostimulation at intensities of 0.1–0.5 MPa for 20 min has been reported, wherein Ca^2+^ influx upon activation of the piezo channel as a result of the stimuli was seen to promote upregulation in calcium/calmodulin-dependent protein kinase II (CaMKII), cAMP Response Element-Binding protein (CREB) and c-Fos,^166^ the latter having been implicated in the induction of neuron excitability.^341,344^

Recently, it was as well noted that exposing natural killer (NK-92) cells to 96.7 MHz SAWs at an applied voltage of 3.6 V_pp_ for 30 min with a 5% duty cycle led to an increase in intracellular Ca^2+^. While the increase, in itself, was insufficient to activate the NK cells alone, which is known to occur through calcium signaling, it was shown that the SAW mechanostimulation led to an approximate 1.5-fold enhancement in the expression of lysosomal-associated membrane protein 1 (LAMP-1)—a marker of NK cell degranulation and hence activation—when applied in conjunction with the addition of phorbol 12-myristate 13-acetate (PMA) that is commonly employed to trigger protein kinase C (PKC) signaling for initiating T-cell activation.^327^

## VI. UNIVERSAL MECHANOTRANSDUCTION MECHANISM

Despite (1) the myriad of mechanosensors that can be activated, (2) the multiple signaling pathways that arise, and (3) the variety of downstream mechanoresponses observed—all as a consequence of the diverse forms of the application of acoustically driven mechanostimulation modes on different cells across a range of frequencies that have been discussed in Sec. IV F, we make the case here for the possibility of a mechanotransduction mechanism that is universal across all such acoustically driven mechanostimulation, at least for both low and high ultrasonic frequencies.

### A. The cell membrane as a universal transducer/effector

The cell membrane, which comprises an asymmetric bilayer containing glycerophospholipids, sphingolipids, cholesterol, and carbohydrates, in addition to high amounts of transmembrane or peripheral proteins,^345,346^ compartmentalizes the cell from its surrounding matrix. As the interface between the microenvironment and the cellular metabolism, it therefore plays an important role in the transduction of mechanical cues into the cell. Specifically, the cell membrane couples directly with effector molecules. These include transmembrane proteins, such as SACs, AJ proteins, GJ proteins,^347–349^ and, mechanosensitive proteins, such as FA proteins.^350^ Any perturbation imposed on the fluidic cell membrane is therefore expected to alter these transmembrane proteins and associated cytoskeletal structures.

These mechanosensory structures on the cell membrane constantly interact with its surrounding microenvironment, allowing the cell to adapt to mechanical stimulation. This is facilitated by tension within the membrane that is generated as a response to the application of an external force on the membrane surface. Membrane tension develops within lipid bilayers and constitutes the basis that defines the structure of all biological membranes. Unlike in pure lipid vesicles, the membrane tension of a cell can vary locally owing to peripheral protein binding, the presence of transmembrane proteins, and interactions with the underlying actomyosin cortex.^351,352^ These interactions provide additional resistance to changes in the membrane area and can constrain such changes to a localized area of the cell. Moreover, they also facilitate mechanotransduction and therefore have the ability to modulate various cellular processes such as endocytosis, exocytosis, cell migration, cytokinesis, mitosis, and intracellular trafficking. For example, changes in membrane tension have been suggested to induce ligand-independent integrin signaling that arises during cell migration.^353^

**FIG. 11.**
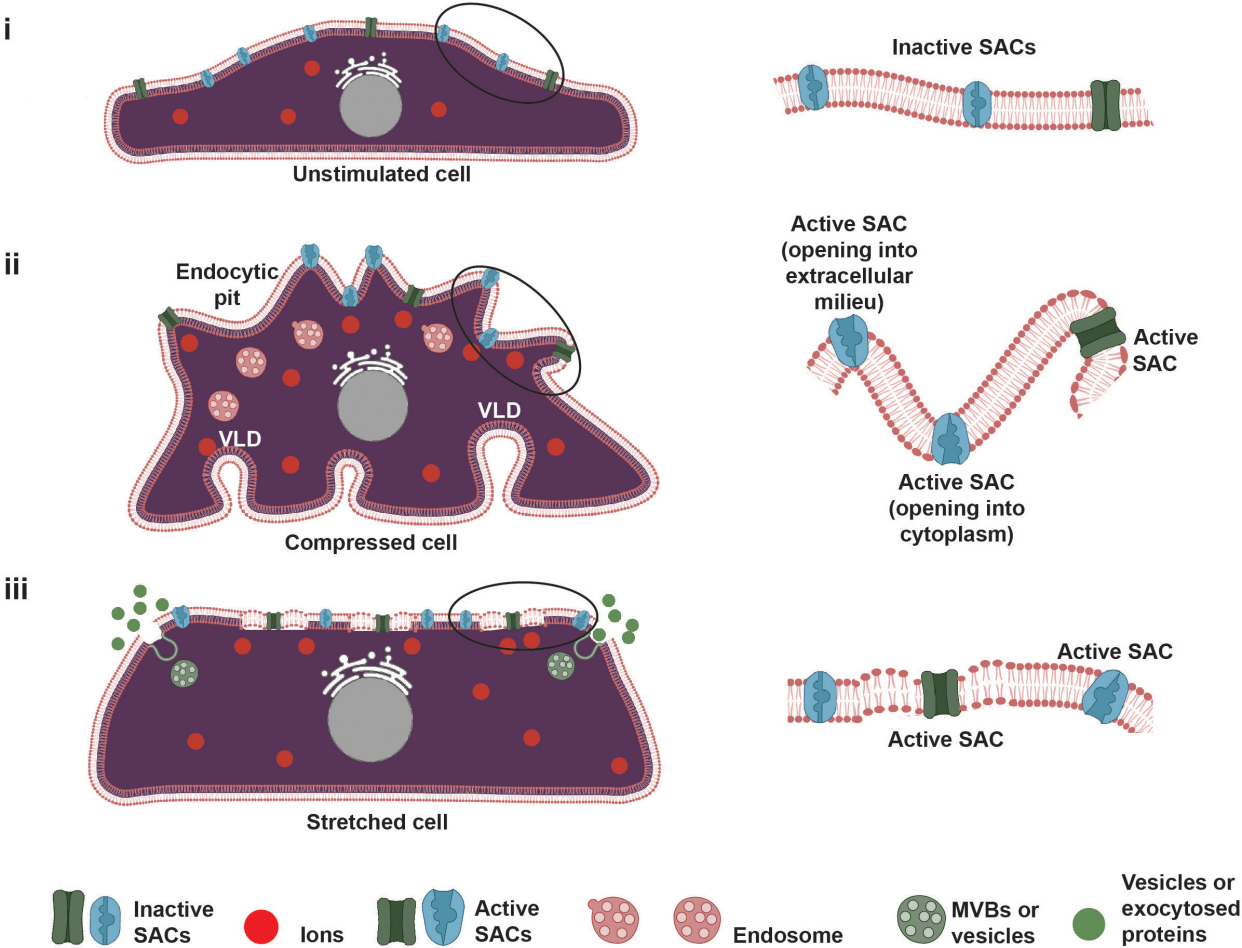
Response of the cell membrane, originally at rest under homeostatic conditions in which the SACs are (i) inactive, to subsequent mechanical stimuli involving (ii) compression or (iii) stretch; the illustrations in the right column are a magnification of the lipid bilayer structure along a representative portion of the cell membrane as indicated by the circle in the illustrations in the left column. Under (ii) compression, the cell membrane folds to form VLDs and endocytic pits to counteract the decrease in membrane tension, leading to the activation of SACs through the FFL gating mechanism illustrated in Fig. 2. Depending on the location of the SAC with respect to the deformation region, it may either open into the ECM to allow ions to bind to the SAC, or into the cytoplasm to release ions into the cell. In contrast, the membrane aberrations and pores that form in cells under (iiii) stretch or tension activate the SACs directly due to the increase in membrane tension, again through the FFL mechanism, leading to an increased propensity for the fusion of multivesicular bodies (MVB) to ease the membrane tension, and resulting in exocytosis and exosome release.

When a cell is mechanically stimulated in a way that changes its membrane tension and shape, the primary response of the cell is to counteract the effect and attempt to restore the membrane tension in order for it to maintain homeostasis, particularly to avoid apoptosis. The actual response depends on the nature and level of the mechanical stimulation since different stimuli alter the membrane tension and shape differently. Mechanical stimuli that constricts the cell leads to membrane compression [Fig. 11(ii)], which reduces the cell size by forming membrane folds of different shapes and sizes with lipid packaging defects in highly curved areas and phosphoinositide clusters.^354–356^ Such changes in membrane curvature are often accompanied by an increase in endocytosis owing to a decrease in membrane tension. To counteract the reduction in membrane tension, vacuole/vesicle-like dilations (VLDs) can also be formed. The formation of these endocytic pits and/or VLDs, in turn, can activate SACs.^54,357^

In contrast, any form of mechanical stimuli which increases the cell area (e.g., stretching the cell) causes both direct activation of SACs through the force-from-lipid model, as well as the flattening of membrane folds, to accommodate the change in tension [Fig. 11(iii)]. The corresponding increase in membrane area involves the use of lipid reserves that, in turn, increases the membrane tension and order, in addition to increasing the propensity for vesicles to fuse to the membrane to increase its area. Both effects result in transient membrane aberrations or perturbations, which also lead to the activation of SACs through the force-from-filament model along with actomyosin contraction.^34,357–359^

In short, all forms of mechanical stimulation—be it static stretch (as a result of tension or shear), compression, or a periodic combination of both effects to constitute more complex dynamical forms of mechanical stimuli such as those due to cavitational-driven poration at low ultrasonic frequencies, or membrane oscillation under SAWs or SRBWs that possess both in-plane and out-of-plane vibrational displacement—lead to some form of disruption to the cell membrane, whether it be to reduce or increase its tension. ^84,87,92,199,360,361^ [While some studies have proposed that mechanical stimuli may induce intra-membrane cavitation, which, in turn, can modulate transmembrane proteins (SACs) and the cytoskeletal structure, they do not explicitly rule out the involvement of the membrane itself since the periodic expansion and contraction of the intra-membrane spaces as a result of such cavitation can itself also lead to stresses on the membrane (Refs. 362 and 363).] Whichever the case, the resultant change in membrane tension drives activation of the SACs, leading to an increase in intracellular Ca^2+^ and triggers downstream effects associated with various Ca^2+^-induced signaling cascades that constitute the cell's mechanoresponse. We note that such mechanosensitivity also applies conversely when the membrane correspondingly relaxes upon cessation of the mechanostimulation.

### B. Ca^2+^ as the key mechanotransduction element

#### 1. Ca^2+^ chemistry

For it to invoke a wide range of biological effects, a metal ion must interact with biological ligands such as proteins, lipids, and carbohydrates by binding to commonly available reaction centers in these biomolecules. Unlike Mg^2+^ and Zn^2+^, which bind with greater affinity to nitrogen ligands, Ca^2+^ has the most affinity for carboxylate oxygen, and, as such, is able bind to these groups in aspartic and glutamic acid, and to the oxygen atoms in Asn (asparagine), Gln (glutamine), Ser (serine), Thr (threonine), and Tyr (tyrosine).^364^

Another relevant parameter that is critical for an efficient second messenger is its binding kinetics. Ca^2+^ binds to and dissociates from a protein a hundred or so times faster than Mg^2+^.^365^ Moreover, its high coordination number (6–8) and often irregular (i.e., protein-induced) coordination geometry allow it to be accommodated into a larger number of proteins.^364^ Ca^2+^-mediated cross-linking is moreover reversible and thus responsive to a change of conditions,^366^ thus allowing cells to easily regulate their concentrations of free and sequestered Ca^2+^ both in time and space by utilizing their calcium stores and calcium-specific ion channels.^367^ Furthermore, Ca^2+^ can either operate within small cellular compartments or act on the entire cytoplasm, with signaling that can last from microseconds to hours, occurring either transiently or in a pulsatile manner.

#### 2. Ca^2+^ mobilization

The underlying principle of Ca^2+^ signaling is the cellular response to a change in its internal Ca^2+^ concentration. In general, the stimulation of a cell leads to an acute increase in its internal Ca^2+^ concentration above the basal level. Upon removal of the stimulus, this concentration returns back to the resting state. The intensity and duration of the stimulus determines the extent of the calcium dynamics. Increases in internal Ca^2+^ concentration can either originate from Ca^2+^ release from intracellular stores and/or Ca^2+^ influx from the extracellular space, assisted by ion channels or transporters on the cell membrane or the ER within the cell.

As large and sustained elevations in internal Ca^2+^ concentration can lead to a variety of deleterious cellular effects, the cell naturally attempts to remove extra Ca^2+^ from the cytoplasm in an effort to return the Ca^2+^ concentration back to its basal level to maintain homeostasis.^368^ This is regulated by the simultaneous interplay of multiple counteracting processes known as Ca^2+^ “on” and “off” mechanisms. During the “on” mechanism, ion channels located on the cell membrane regulate the supply of Ca^2+^ from the ECM, and ion channels on the ER from the intracellular storage space. In the “off” mechanism, cells remove Ca^2+^ from the cytoplasm either by storing extra Ca^2+^ in its intracellular stores such as the ER or by effluxing Ca^2+^ back into the extracellular milieu through Ca^2+^ATPases on the cell membrane and the ER through activation of its ion channel receptors, such as inositol 1,4,5-trisphosphate (IP3R) and ryanodine (RyR), along with ion exchangers that utilize gradients of other ions to provide the energy to transport Ca^2+^ out of the cell.^17^

**FIG. 12.**
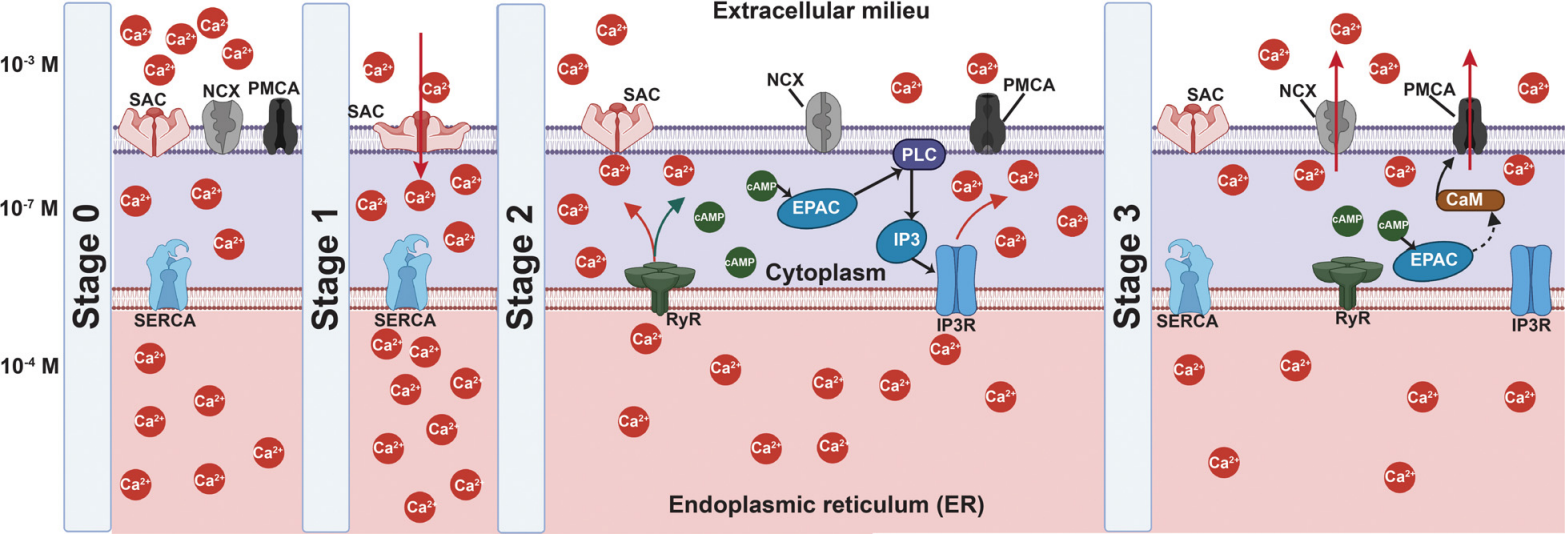
Calcium mobilization stages and the various signaling cascades they induce. Stage 0: The cytoplasmic Ca^2+^ level is maintained at relatively low levels ( $∼10^{-7}$ M) in resting cells by influx and efflux through plasma membrane Ca^2+^ ATPase (PMCA) pumps and Na^+^/Ca^2+^ exchangers (NCX) along the cell membrane and via smooth endoplasmic reticular Ca^2+^ ATPase (SERCA) transporters along the ER membrane. Stage 1: With a change in membrane tension, SACs are activated, leading to an influx of Ca^2+^ into the cytoplasm that increases the cytoplasmic Ca^2+^ levels. This is rapidly restored by activation of the SERCA pumps that transport the Ca^2+^ ions into internal stores, mainly the ER. Stage 2: The increase in intracellular Ca^2+^ is followed by slow release of Ca^2+^ back into cytoplasm through calcium-induced calcium release (CICR) primarily from the ER via Ca^2+^-sensitive ryanodine receptors (RyR), accompanied by an increase in intracellular cAMP that activates the Epac signaling cascade. This, in turn, triggers the formation of the phospholipase C (PLC) pathway to activate the IP3 receptor (IP3R) that stimulates further release of Ca^2+^ from the ER store. Stage 3: The Ca^2+^ that is released from the ER is slowly effluxed back into the ECM through NCX. Meanwhile, Epac activates Ca^2+^/calmodulin (CaM) to trigger Ca^2+^ efflux via PMCA back into the ECM.

At highly elevated Ca^2+^ levels, quick restoration of the cytosolic Ca^2+^ concentration to its basal level occurs through the movement of intracellular Ca^2+^ via the SERCA pump that mediates its redistribution within the ER (Fig. 12).^369^ As such, the influx of Ca^2+^ from the extracellular milieu can initiate Ca^2+^-induced Ca^2+^ release (CICR) and RyR activation when the stores' Ca^2+^ content exceeds normal physiological values (i.e., “store overload”),^370^ releasing Ca^2+^ primarily into the cytosol where it may be released back into the extracellular milieu.^369^ The RyR-mediated Ca^2+^ release, in turn, activates IP3R-mediated Ca^2+^ release from the ER.^371^ Such Ca^2+^ release from the ER could then enhance the Ca^2+^ efflux activity of sodium/calcium exchangers (NCX) and plasma membrane calcium ATPase (PMCA) pumps.^372^ This process by which a cell maintains its internal Ca^2+^ concentration spatiotemporally through various receptors and ion channels is known as Ca^2+^ mobilization.

Such a unique ability of the cellular machinery to spatiotemporally modulate intracellular Ca^2+^ concentration renders Ca^2+^ an ideal messenger for mechanotransduction. The versatility of Ca^2+^ signaling, which is evidenced from the fact that Ca^2+^ controls almost every aspect of cellular activity from its infancy to death, and its diversity in directing different activities depending on the cell type stemming from non-transcriptional events (phosphorylation/dephosphorylation events to activate proteins) or transcriptional effects,^373^ arises from its potential to interact with a wide range of molecules, including cAMP, nitric oxide, phosphatidylinositol-3-OH kinase, and MAPK. As seen in Fig. 4, these interactions trigger a multitude of signaling cascades, such as the phosphatidyl inositol (PI), phospholipase C (PLC), inositol triphosphate (IP3), protein kinase C (PKC), and calmodulin (CaM) or calmodulin kinase II pathways, and can crosstalk with G protein-coupled receptor (GPCR) signaling [e.g., protein kinase A (PKA) and receptor tyrosine kinase (RTK)] pathways to control almost every cellular activity, from short-term effects (e.g., gene transcription, and, cell contraction and secretion) to longer-term effects regulating cell development and regeneration (e.g., cell proliferation, migration, differentiation, apoptosis, and necrosis).

Interestingly, downstream effectors of Ca^2+^ signaling can also, in turn, regulate Ca^2+^ mobilization. For instance, RyR-mediated Ca^2+^ release from the ER is accompanied by activation of adenylyl cyclase (AC) and an increase in cAMP, which then initiates the Epac-mediated signaling cascade. RyR activation can also trigger Epac directly that initiates PLC signaling to result in the formation of IP3 that initiates IP3R-triggered Ca^2+^ release from the calcium store.^374^ Essentially, any signaling cascade triggering the formation of IP3 is able to activate IP3R-triggered Ca^2+^ release from the ER, whereas increases in intracellular Ca^2+^ alone triggers RyR activation.^371^ Whichever the case, Ca^2+^ release from the ER enhances the Ca^2+^ efflux activity of NCX^372^ while Ca^2+^ efflux through plasma membrane calcium ATPase (PMCA) is regulated by calmodulin, PKC, and PKA (products of the cAMP–Epac pathway).^375^ In short, intracellular Ca^2+^ not only regulates cellular activities but also its own mobilization, and, as such, initiates multiple signaling cascades at any stage of the mobilization.

A possible explanation for this exquisite behavior could be the plethora of Ca^2+^-binding proteins (which regulate Ca^2+^ homeostasis to activate different Ca^2+^ signaling cascades that determine downstream cell response and fate) that exist, and the wide range of affinities they possess (which vary by 10^6+^-fold), depending on their location and function.^376^ Based on a study conducted on effectors of calcium-binding proteins such as Ca^2+^/calmodulin-dependent protein kinase II (CAMKII) and PKC, different cellular activities were observed to be determined by the magnitude and frequency of the Ca^2+^ oscillation:^377–380^ brief Ca^2+^ oscillations/spikes evoke simpler cellular activities such as cytoskeletal rearrangement, vesicle trafficking (including neurotransmitter release), and contraction,^17^ whereas complete Ca^2+^ mobilization has the propensity to trigger more complex processes such as differentiation^381–383^ and angiogenesis,^384–386^ or even oocyte activation.^387,388^

### C. Acoustic stimulation

**FIG. 13.**
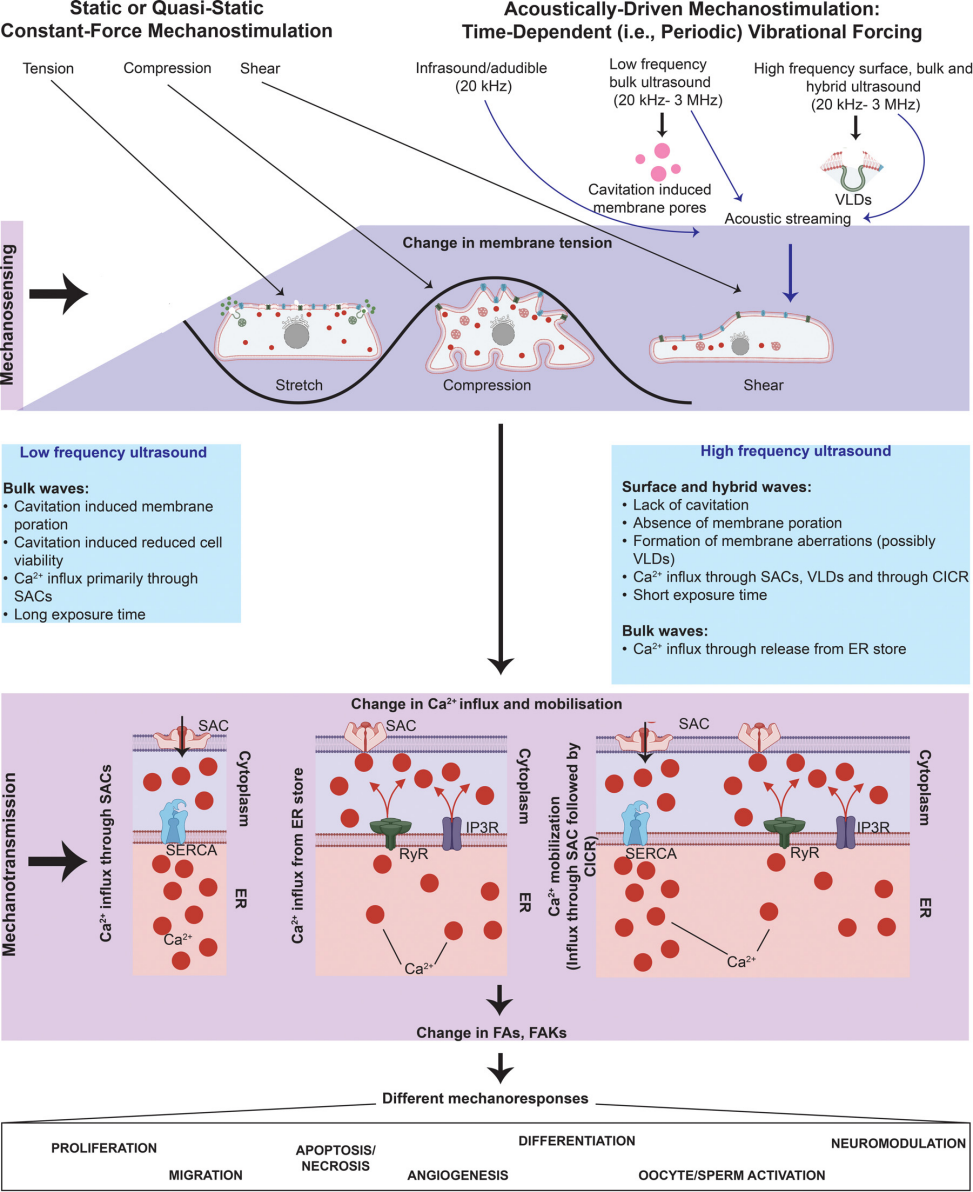
Proposed universal mechanism that underpins a cell's response to different forms of mechanostimuli, wherein the cell membrane plays a central role as a universal transducer/effector, and the second messenger Ca^2+^ as a key mechanotransduction mechanism. Static or quasi-static constant force mechanostimuli in the form of compression, stretch, or shear directly alter membrane fluidity to drive influx of Ca^2+^ into the cell that, in turn, trigger different downstream signaling pathways to elicit a variety of mechanoresponses. More complex dynamical forms of mechanostimuli, such as those driven acoustically, invoke changes to the membrane tension indirectly by either inducing cavitation, perturbations or aberrations to the membrane, or the generation of acoustic streaming in the cell media. They nevertheless lead to similar mechanoresponses given that these effectively result in compression, stretch, or shear to the cell, or more complex combinations thereof. Where mechanoresponses differ, however, is a consequence of how Ca^2+^ is regulated and hence mobilized within the cell in response to these mechanotransductive processes.

All forms of acoustic stimulation, irrespective of frequency, are able to modulate intracellular Ca^2+^ to drive its mobilization within the cell in one way or another by altering the tension of the cell membrane, as described in Sec. VI A and illustrated in Fig. 13. At lower (Hz- to kHz-order) frequencies where cavitation is prevalent, this could occur either directly through the formation of membrane pores or via activation of SACs such as the TRPV channels. At higher (MHz-order) frequencies where cavitation is suppressed, the influx of Ca^2+^ is primarily driven through transient formation of VLDs or the activation of piezo channels (the involvement of voltage-gated ion channels has also been implicated at GHz frequencies^389^) with Ca^2+^ efflux from the ER back into the extracellular milieu being dependent on, or, in some instances,^390^ independent of, the extracellular Ca^2+^ concentration.

Differences in Ca^2+^ mobilization between the frequency regimes can, nevertheless, provide an explanation for why prolonged exposure times are required with low frequency acoustic stimulation whereas significantly shorter durations are sufficient to invoke similar cellular responses at high frequencies, particularly that with SRBW mechanostimulation. Moreover, given the different stages of Ca^2+^ mobilization that exist, a plethora of signaling cascades can be evoked, which can explain the distinct mechanoresponses observed for the various forms of acoustic stimuli on different cell types, or even that observed with the same acoustic stimuli but under different excitation parameters. As such, a deeper understanding of the Ca^2+^ mobilization induced for each specific case is required to design and develop more effective and targeted therapeutic strategies.

## VII. SUMMARY

Sound wave excitation offers a considerably broader means of mechanically stimulating cells. Unlike steady forms of mechanical loading (e.g., compression, tension, or shear) applied on the cells under constant loads, either statically or quasi-statically at frequencies of several Hz, dynamic vibrational stimuli can be excited in distinct forms of bulk, surface, or hybrid acoustic waves across a broad amplitude and frequency spectrum to not only evoke far richer, albeit more complex, cellular responses, but to also allow tunability for a diverse range of applications.

Low frequency (Hz to kHz) excitation, for example, has longer characteristic length scales over which the acoustic energy decays, thus allowing greater penetration, particularly through skin and tissue, and thereby enabling their use for *in vivo* applications. A corollary of the use of low frequency mechanostimulation, however, is the existence of cavitational effects, which, besides exerting considerable stress on cells, can also induce poration along the cell membrane. The larger intensities and prolonged exposure durations that are typically required with low frequency acoustic excitation can also introduce considerable thermal effects that could potentially induce the cells to produce heat shock proteins.

These effects can be circumvented by operating at higher frequencies (MHz order and beyond), whose wavelengths are more closely matched to the characteristic cell dimension and hence allows for localization of the mechanostimulation signal, thereby reducing the intensity required to elicit the same effect. This reduction in power and hence heating as a consequence of absorption of the acoustic energy, together with the diminished propensity for cavitation, allows significantly greater retention in cell viability. The considerably shorter penetration depths associated with high frequency excitation however precludes their use for *in vivo* applications unless implanted at the local site to be targeted. Nevertheless, the much shorter exposure durations that have been demonstrated with the use of surface and hybrid waves—which have been shown to facilitate both efficient poration-free cellular uptake as well as exosome release in addition to stem cell differentiation and endothelial barrier modeling—together with the possibility for parallelization of the chipscale technology can be appealing for large-scale cellular engineering.

In addition to providing an overview of the advances of acoustically driven cell mechanostimulation across these frequencies to date, and their utility for various applications, particularly in, but not limited to, regenerative therapeutics and nanomedicine, we postulate that the mechanoresponses stemming from such stimuli, particularly those driven at ultrasonic frequencies across 10 kHz–1 GHz, arise from a universal mechanotransductive mechanism regardless of the considerable differences between the underlying mechanostimulation modalities (e.g., constant force, static or quasi-static stimuli such as compression, tension or shear, or, more complex dynamic stimuli involving time-dependent vibrational forcing in the form of bulk, surface or hybrid acoustic waves) across a wide range of frequencies.

Broadly, the cellular response to acoustic forcing, and, mechanostimulation, in general, arises as an outcome of the cells' attempt to maintain both membrane and Ca^2+^ homeostasis. We posit that the cell membrane plays a central mechanosensory role and that the mechanoresponses that are observed can be attributed to the effect of the mechanical cue, whatever its form, imparted on the cell membrane. Be it static compression or tension (stretching) of the membrane, or a dynamic combination of these constituents as a consequence of the time-dependent, i.e., periodic, vibrational forcing associated with the acoustic excitation, the resultant membrane aberrations (e.g., VLDs) or pores that arise lead to a change in the membrane tension that then drives the activation of SACs. It is primarily these SACs, together with auxiliary gating mechanisms such as other transmembrane proteins (e.g., integrins) and cell junctional proteins (e.g., connexins and cadherins), that facilitate the transmission of the imparted mechanical force through the rest of the cell via its cytoskeletal network to trigger a cascade of downstream molecular signaling events.

More specifically, we observe the second messenger Ca^2+^ to play a central role in maintaining cellular homeostasis in response to the mechanical insult. In the first Ca^2+^ mobilization stage, Ca^2+^ influx into the cell facilitated by the SAC and cell junctional proteins trigger the activation of a series of signaling cascades that include Rho–ROCK signaling and ESCRT pathways. In the second stage of Ca^2+^ mobilization, the aforementioned influx of Ca^2+^ into the cell as a consequence of the mechanical load is regulated by channeling it into internal stores primarily through SERCA pumps or returning it to the extracellular milieu through calcium transporters on the membrane. Upon Ca^2+^ overload in the internal store, Ca^2+^ is further released through RyR activation, which results in the production of other second messengers such as cAMP and IP3. These second messengers further initiate other signaling cascades (e.g., the Epac1-Rap1 pathway) to facilitate activation of transcription factors, including c-Jun, c-Fos, or ERK1/2, which regulate a diverse set of cellular activities such as cell proliferation, migration, and exosome generation, in addition to other cell-specific processes such as stem cell differentiation, endothelial barrier modulation, neuromodulation, and oocyte activation.

The translation of mechanostimulation to practical living systems for clinical therapeutic applications, however, remains a challenge. Unlike their *in vitro* counterparts, *in vivo* systems are considerably more complex due to the lack of appropriate models and means to study the collective behavior arising from the number of different cell types that are simultaneously stimulated, and the multitude of mechanoresponses over widely varying response timescales that can arise.^391^ As such, there is, at present, insufficient conclusive evidence for coordinated mechanotransduction *in vivo*, and a thorough understanding of the underlying mechanisms that are involved is still elusive.^392,393^ In addition, the vast number of systems and protocols associated with multicellular bodies make it significantly more difficult to compare and interpret the data required to upscale these platforms for practical applications. Nevertheless, a better understanding of the *in vitro* mechanotransduction mechanisms stemming from that discussed in this review would hopefully constitute a start in advancing the technology toward clinical translation.

## Data Availability

Data sharing is not applicable to this article as no new data were created or analyzed in this study.
